# Azole γ‐Peptides Helix Switching via Heterocycle Substitutions

**DOI:** 10.1002/anie.9299244

**Published:** 2026-07-16

**Authors:** Samantha Chaise, Claude Didierjean, Audrey Gacogne, Maxime Fillaudeau, Young Kee Kang, Aurélien Lebrun, Jean‐Louis Bantignies, Dominique Housset, Muriel Amblard, Ludovic T. Maillard, Baptiste Legrand

**Affiliations:** ^1^ IBMM UMR5247 Univ. Montpellier CNRS ENSCM Montpellier France; ^2^ CRM2 Université de Lorraine CNRS Nancy France; ^3^ Department of Chemistry Chungbuk National University Cheongju Chungbuk Republic of Korea; ^4^ PAC Chimie Balard Univ. Montpellier CNRS ENSCM Montpellier France; ^5^ L2C UMR5221 Univ. Montpellier CNRS Montpellier France; ^6^ IBS Université Grenoble Alpes CEA CNRS Grenoble France

**Keywords:** γ‐peptides, foldamers, helix switching, heterocycles, stereoelectronic control

## Abstract

Precise control of peptide backbone folding through non‐covalent interactions remains a major challenge in foldamer design. In this work, we demonstrate that heteroatom substitutions program conformational switching in azole γ‐peptides by tuning intrinsic stereoelectronic effects within heterocyclic γ‐amino acids. Conformationally constrained thiazole‐ and oxazole‐based γ‐amino acids were designed to adopt conformations driven by either a *C*_9_ or a *C*_7_ intramolecular H‐bond depending on key 1,4‐X···O interactions (X = S or N). We previously showed that thiazole‐based oligomers form a well‐characterized canonical 9‐Helix. Here, permutation of the sulfur and nitrogen atoms within the heterocycle induces a stretched helical structure with alternating *C*_7_‐turns and residues in extended conformations. This unusual topology arises from competition between seven‐membered intra‐residue H‐bonds and attractive S···N electrostatic‐chalcogen interactions. Reversing this effect through an S→O substitution affords oxazole‐derived oligomers that adopt a stable 7‐Helix stabilized by a continuous seven‐membered H‐bond network in solution. These findings show that simple heteroatom permutation or substitution allows control over heterocyclic γ‐peptide folding, thereby expanding opportunities for foldamer design in molecular recognition, catalysis, and biomedical applications.

## Graphical abstract

Heterocycle substitution in azole‐based γ‐amino acids enables control over the helical shape of heterocyclic γ‐peptides. S↔N permutation in thiazole residues switches a canonical 9‐helix into a flexible helix exhibiting discontinuous hydrogen bonding. Further S→O substitution in oxazole derivatives directs folding toward a stable 7‐helix in solution.

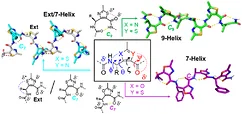

## 1 Introduction

Five‐membered heterocycles are ubiquitous motifs found in both natural products [1, 2, 3, 4, 5] and synthetic compounds [6, 7], including catalysts [8], dyes [9], and pharmaceuticals [10, 11]. Their conformational rigidity and ability to engage in various non‐covalent interactions such as hydrogen bonding, π‐stacking, and dipolar effects, enable fine control over local geometry and strongly influence molecular properties and bioactivity [12, 13, 14, 15]. Within peptides, azole‐containing residues are particularly effective at imposing conformational constraints [16, 17], enhancing proteolytic stability [18, 19], and enabling metal coordination [20, 21, 22, 23], thus motivating their incorporation into peptidomimetics and foldamers [24, 25, 26, 27, 28, 29, 30, 31].

**FIGURE 1:**
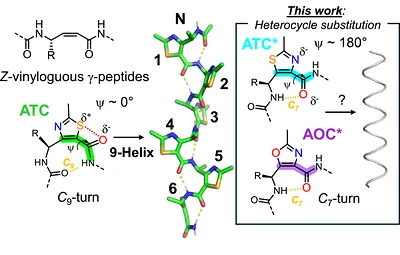
Background for this study. ATC: 4‐Amino‐(methyl)‐Thiazole‐2‐Carboxylate‐derived γ‐amino acid; ATC*: isomer of ATC in which N and S were permuted within the thiazole ring; AOC*: 5‐Amino‐(methyl)‐Oxazole‐4‐Carboxylic acid, the oxazole analogue of ATC* in which S is replaced by O. The asterisk notation distinguishes the permuted/substituted variants from the parent ATC thiazole‐based γ‐amino acid described in the original work on ATC [32]. ATC adopts a *C*_9_‐turn and oligomers form a 9‐Helix (green), whereas the ATC* and AOC* heterocyclic γ‐amino acids form *C*_7_‐turns [33]. Hydrogen bonds and electrostatic interactions are shown as yellow and red dotted lines, respectively.

Building on this concept, we previously introduced a class of heterocyclic γ‐amino acids based on a thiazole core, known as ATC (Figure 1) [32, 34, 35]. These residues, formally related to *Z*‐vinylogous γ‐amino acids through the α‐β unsaturation, are preorganized into *C*_9_‐turn stabilized by a 9‐membered H‐bond and an attractive 1,4‐S···O stereoelectronic interaction involving the thiazole and the carboxamide. ATC oligomers adopt a right‐handed 9‐helix, a scaffold that has been successfully leveraged for antimicrobials design [36], inhibition of amyloid aggregation [37], multivalent recognition [38, 39], and organocatalysis [40, 41]. Despite these advances, the broader conformational landscape of γ‐peptides remains underexplored. Notably, on the basis of *ab initio* molecular orbital (MO) theory, Hofmann and coworkers predicted that Z‐vinylogous γ‐peptides could access two stable helical states: the 9‐Helix observed for ATC oligomers (Figure 1) and a 7‐Helix [42]. In this work, we investigated whether heterocycle variation could direct conformational switches and provide access to alternative folds, thereby expanding the conformational space of azole γ‐peptides.

## 2 Results and Discussion

**FIGURE 2:**
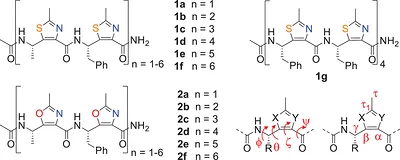
ATC* and AOC* sequences. Nomenclature of atoms and torsion angles.

**FIGURE 3:**
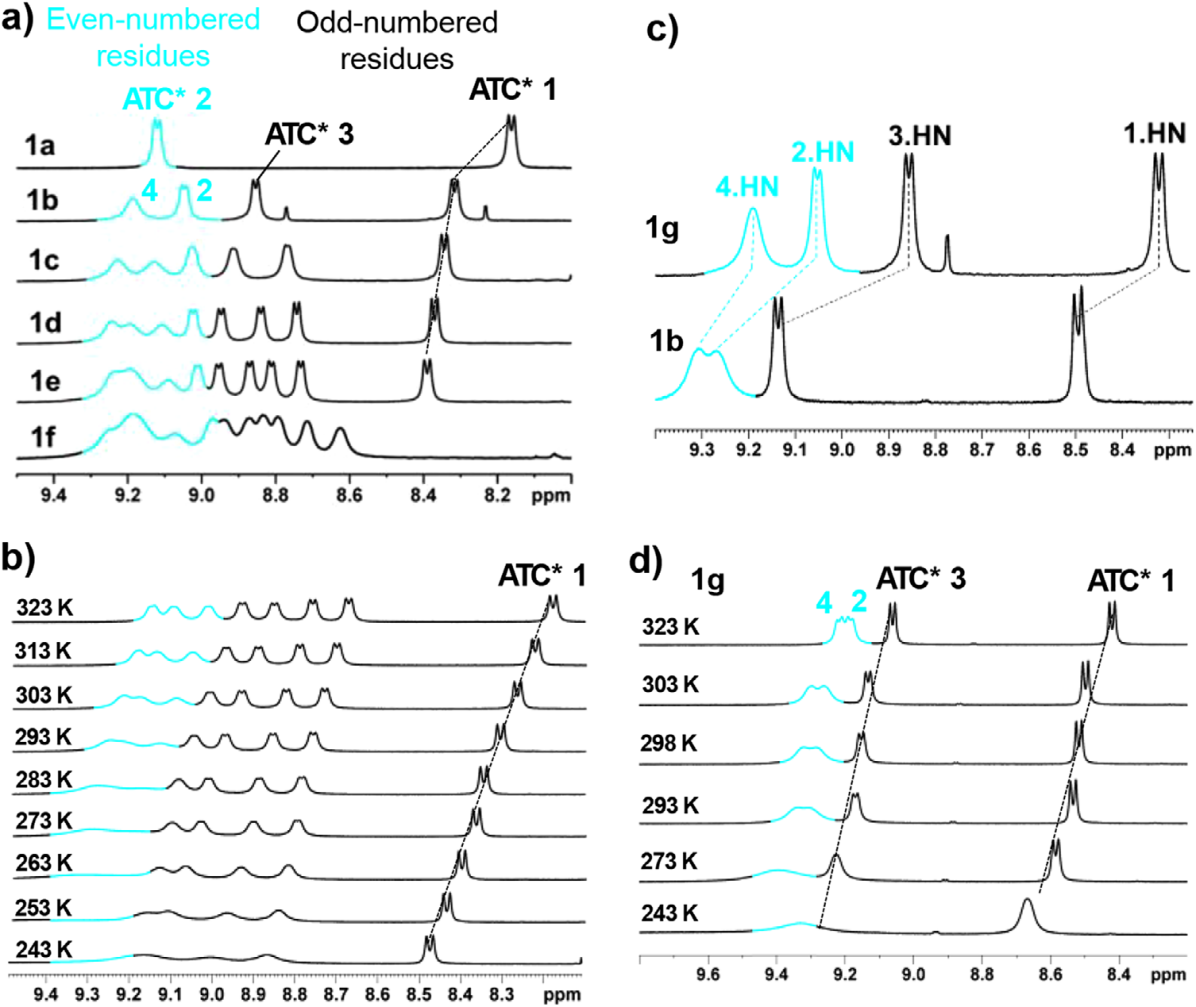
(a) HN regions in the ^1^H NMR spectra of ATC* oligomers **1a–f** in CDCl_3_ at 298 K, showing the two distinct amide proton populations. Resonances of the amide protons of the even‐numbered residues were colored in cyan. (b) NMR temperature‐dependent behaviors of amide protons in **1d**. (c) Comparison of the HN regions in the ^1^H NMR spectra of ATC* tetramers **1b** and **1g**. (d) NMR temperature‐dependent behaviors of amide protons in **1g**.

**FIGURE 4:**
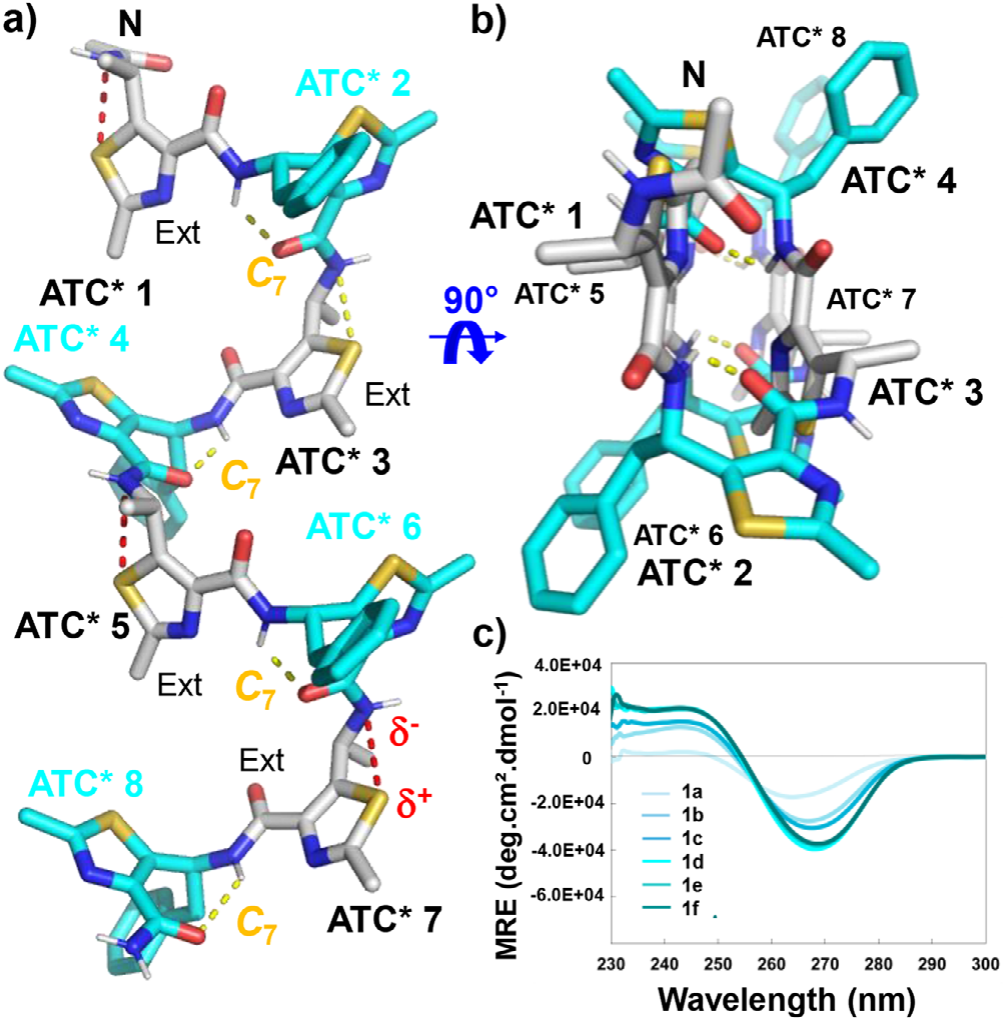
(a) Longitudinal and (b) axial views of the MicroED structure of the left‐handed helix of **1d** alternating *C*_7_‐turns (in cyan) and extended segments (in grey). Hydrogen bonds and electrostatic interactions are shown as yellow and red dashed lines, respectively. (c) CD profiles of oligomers **1a–f** at 100 µM in CHCl_3_ at 20°C.

**FIGURE 5:**
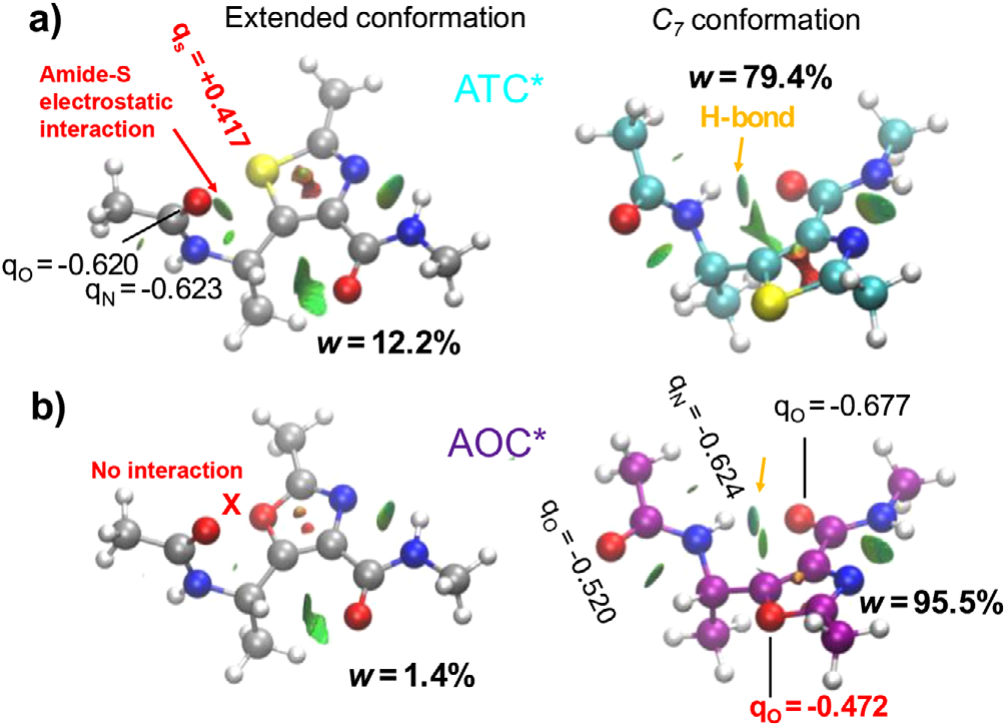
Intraresidue interactions within (a) ATC* and (b) AOC* monomers in the extended (Ext) and *C*_7_ conformations. Noncovalent interactions were analyzed by NCIplot and partial charges were calculated using Gaussian 09. The red arrow highlights the attractive electrostatic interaction between sulfur and the preceding amide bond, while the yellow arrow indicates the seven‐membered hydrogen bond. Boltzmann populations from conformational analysis (*w*, in %) are indicated for each conformer.

We recently introduced two novel heterocyclic γ‐amino acids, ATC* and AOC* (Figure 1), designed as protein‐turn mimetics [33, 43]. ATC* is a thiazole‐based isomer of ATC in which the nitrogen and sulfur atoms are permuted, while AOC* is the corresponding oxazole analogue. While ATC folds into *C*_9_‐turns, both ATC* and AOC* form *C*_7_‐turns stabilized by seven‐membered intramolecular hydrogen bonds involving the NH donor and the carbonyl acceptor within a single residue. These structural features suggest that ATC* and AOC* oligomers may exhibit a shifted backbone conformational preference. To explore this possibility, we first focused on ATC* oligomers. *N*‐Fmoc‐ATC*‐OH monomers bearing either a methyl or a benzyl side chain on the γ‐carbon, were synthesized following our previously reported procedures [43]. Oligomers **1a–f** (Figure 2), ranging from dimer to dodecamer, were then assembled by SPPS on Rink amide resin using DIC/Oxyma Pure as coupling reagents. First, NMR spectra were typically recorded at 2 mM concentration in CDCl_3_ at 298 K on oligomers **1a‐f** (Figures 3 and S8). Regardless of the oligomer length, the ATC* oligomers exhibited two sets of HN resonances (Figure 3a), i.e. broad downfield signals at 9.0–9.3 ppm (in cyan) assigned to the even‐numbered residues bearing benzyl side chains while the sharp resonances below 9.0 ppm corresponded to the odd‐numbered residues carrying methyl groups. Upon decreasing the temperature, selective broadening of the HN resonances was observed, indicative of an intermediate‐timescale conformational exchange that may arise from conformational heterogeneity (Figure 3b). Unfortunately, these broad signals prevented complete chemical shift assignments beyond the tetramer and the collection of the full set of NOE correlations required to determine convergent ensemble NMR structures. Nevertheless, we unambiguously identified long‐range 1.Hτ‐3 and 4.Hγ NOE correlations for tetramer **1b** consistent with the spatial proximity between the thiazole methyl group of residue i and the thiazole ring of residue i + 3 (Figure S9). To assess whether this conformational behavior depended on side chain identity, we synthesized ATC* tetramer **1g**, in which all residues bear benzyl groups (Figure 2). The ^1^H NMR spectrum of **1g** closely resembled that of **1b** with comparable ^3^ *J*(HN–Hγ) coupling constants values around 8 Hz (Table S20), also displaying two distinct sets of HN linewidths that broaden as the temperature decreases (Figures 3c,d). Thus, the conformational preference of ATC* oligomers appear to be primarily governed by the thiazole backbone rather than by the nature of the side chains. Importantly, octamer **1d** produced microcrystals (< 1 µm) upon slow evaporation of a DCM/Et_2_O mixture, which were suitable for microcrystal electron diffraction (MicroED) analysis (Table S1) [44, 45]. The MicroED structure of **1d** was then optimized at the PCM M06‐2X/6‐31G(d) level of theory in chloroform prior to analysis (Figure 4a,b). The solid‐state structure revealed an unusual left‐handed stretched helix with 5 residues per turn and a pitch of about 13 Å. The helix displayed an elliptical cross section with minor and major axes of approximately 3.8 Å and 7 Å, respectively. This architecture arises from an alternating pattern of *C*_7_‐turns stabilized by seven‐membered pseudorings at even‐numbered residues and extended conformations at odd‐numbered residues. This discontinuous hydrogen bond network may explain the two different sets of HN resonances observed on the NMR spectra of ATC* oligomers. In the crystal, each helix is tightly packed against four symmetry‐related helices with opposite directions, engaging in multiple intermolecular interactions (Table S2 and Figure S1). In solution, ^1^H NMR spectra of **1d** recorded at 200 µM and 2 mM were comparable, indicating that no self‐assembly may occur within this concentration range (Figure S10). To gather additional evidence regarding the folding of ATC* oligomers in solution, FT‐IR and CD experiments were performed (Figures 4c and S15). In the FT‐IR spectra, both amide A (ν(NH)) and amide I (ν(CO)) vibrations are classically downshifted when involved in hydrogen bonding. ATC* oligomers **1a–f** displayed ν(NH) bands at ∼3390 cm^−1^ and ∼3320 cm^−1^, corresponding to free and H‐bonded NH groups, respectively, while ν(CO) bands were at ∼1672 cm^−1^ and ∼1652 cm^−1^ for free and H‐bonded C = O groups. Integration of band intensities revealed nearly equal populations of free and H‐bonded amides consistent with the solid‐state structure of **1d** (Figures S16–S21; Table S36). CD spectra of heterocyclic γ‐peptides are typically dominated by a strong Cotton effect arising from the heterocycles [32]. Unlike the right‐handed ATC 9‐Helix, which previously showed a strong positive maximum around 260 nm, ATC* oligomers exhibited a negative maximum at ∼268 nm (Figure 4c). Notably, the CD profiles showed a chain‐length dependence, with the maximum shifting from 264 to 268 nm and the intensity increasing up to ∼39 500 deg·cm^2^·dmol^−^^1^ as the oligomer length increases. Taken together, these results indicate that ATC* adopts a left‐handed 7‐helix in the solid state, whereas in solution it displays limited conformational stability, as evidenced by the conformational exchange processes observed by NMR. To rationalize the intraresidue interactions that may contribute to the conformational duality between the *C*_7_ and extended conformations, we performed DFT calculations, including natural bond orbital (NBO) analyses and evaluation of electrostatic interaction energies at the M06‐2X/def2‐TZVP level on Ac‐ATC*‐NHMe using the Gaussian 09 program (Figures 5a, S2–S6 and Tables S3–S5) [46]. The *C*_7_ conformation is primarily stabilized by the seven‐membered intramolecular hydrogen bond. In the extended conformation, the thiazole sulfur carries a positive partial charge of +0.417 e and engages in favorable electrostatic interactions with the adjacent negatively charged carbonyl oxygen (q(*O*) = ‐0.620 e) and amide nitrogen (q(*N*) = ‐0.623 e), resulting in a total electrostatic stabilization (Δ*E*) of ‐11.1 kcal.mol^−1^ in chloroform (Figures 5a and S2; Table S3). NBO analysis also reveals an intra‐residue chalcogen interaction between thiazole sulfur and amide nitrogen, contributing to the stabilization of the extended state (Table S4). The competing interactions between attractive electrostatic‐chalcogen interactions and *C*_7_ hydrogen bonding within ATC* monomer may explain the intrinsic conformational dynamics observed in solution. Based on these results, we hypothesized that replacing sulfur with a more electronegative oxygen, in oxazole‐based γ‐amino acids (AOC*), will favor the *C*_7_‐turn formation and ultimately a regular 7‐Helix. NBO analysis supports this hypothesis, showing that the seven‐membered hydrogen bond in the C7ax conformation of the AOC* monomer was 3.2 kcal mol^−^^1^ stronger than in ATC* (Table S4), with the C7ax population increasing accordingly from 79.4% to 95.5% (Figures 5 and S3; Table S5).

**FIGURE 6:**
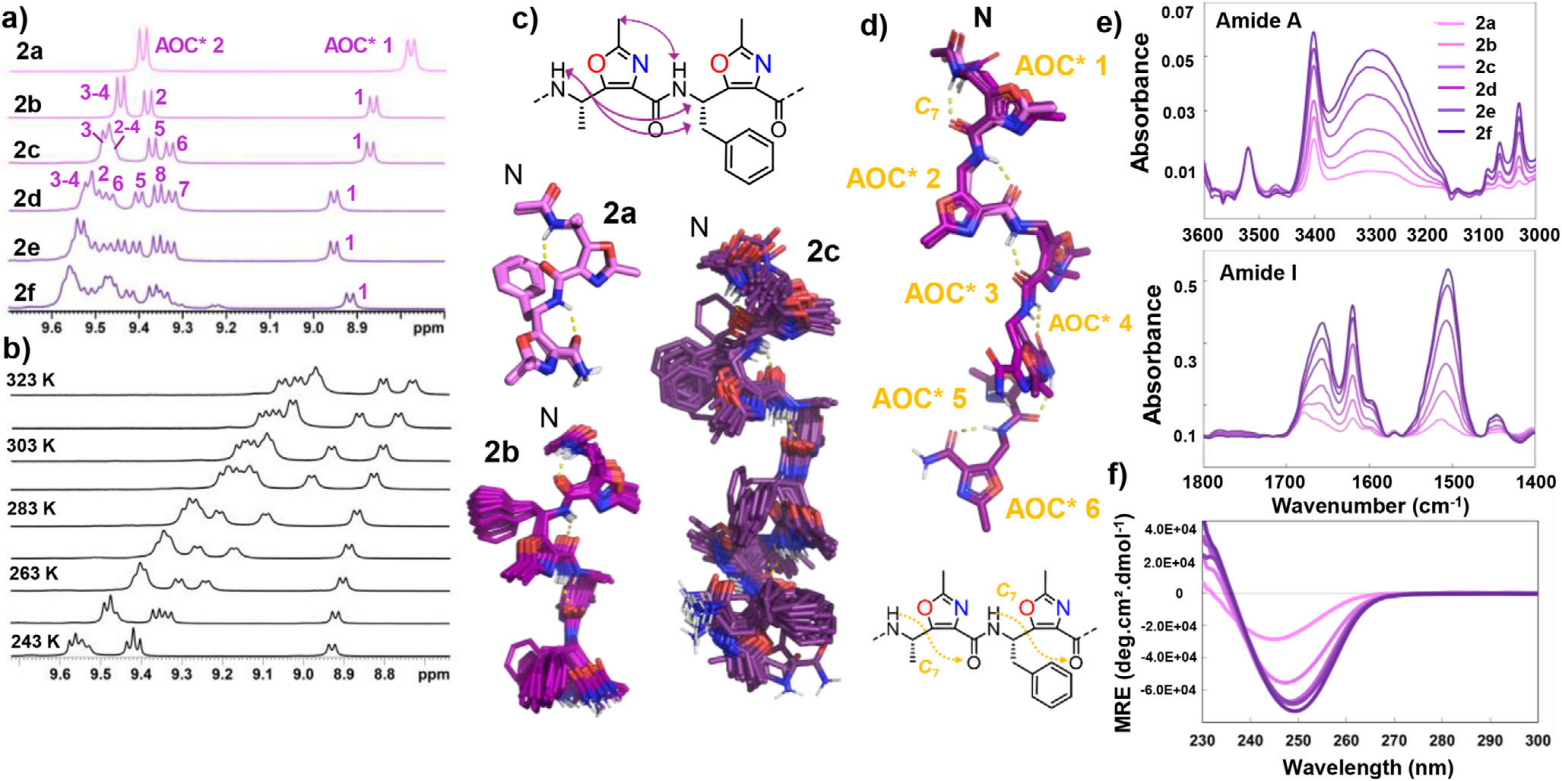
NMR, FT‐IR, and CD characterization of AOC* oligomers **2a‐f** in solution. (a) HN regions in the ^1^H NMR spectra of AOC* oligomers **2a–f** in CDCl_3_ at 253 K. (b) Temperature‐dependent resonances of amide protons in **2c** (243–323 K). (c) Characteristic NOE correlations of the AOC* oligomers and superimposition of the 20 lowest‐energy NMR solution structures of **2a–c**. (d) Superimposition of the DFT‐optimized lowest‐energy NMR structures of **2a–c** obtained at the PCM M06‐2X/6‐31G(d) level of theory in chloroform. Typical hydrogen‐bond pattern stabilizing the AOC* oligomer 7‐Helix. (e) Amide A and amide I regions of FTIR spectra of oligomers **2a–f** at 1 mM in CHCl_3_ at 20°C. (f) CD profiles of oligomers **2a–f** at 100 µM in CHCl_3_ at 20°C.

To further investigate, AOC* oligomers **2a‐f** (Figure 2) were synthesized and subjected to extensive structural analysis. Although no crystals suitable for X‐ray diffraction could be obtained, NMR spectra displayed sharp and well‐dispersed signals with downfield resonances up to 9 ppm (Figure 6a), enabling extensive structural investigations in solution. Upon cooling, HN resonances sharpened slightly and shifted downfield to 9.6 ppm consistent with a discrete conformer with stable hydrogen bonding (Figure 6b). ^3^ *J*(HN–Hγ) coupling constants were measured to probe local backbone conformations (Table S20). While ^3^ *J*(HN–Hγ) average values around 7.5‐8.0 Hz in ATC* sequences were not conclusive, AOC* oligomers exhibited large couplings of ∼9‐10 Hz, indicative of an anti‐periplanar arrangement between HN and Hγ protons. In this context, nearly all resonances were assigned for AOC* oligomers up to the octamer, while longer sequences suffered from signal overlap. Unlike ATC* oligomers, numerous non‐sequential NOEs were detected, including HNi‐Hγ(i+1), HNi‐Hδ(i+1) and Hτi‐HN(i+1) correlations, suggesting compact and periodic structures (Figure 6c). Complete sets of NOEs were used as restraints for solution structure determination of AOC* dimer **2a**, tetramer **2b**, and hexamer **2c**, using simulated annealing in AMBER 22 [47]. The calculations were performed using 61, 96, and 135 unambiguous distance restraints for the dimer, tetramer, and hexamer, respectively (Table S21). The resulting lowest‐energy structures exhibited a left‐handed 7‐Helix stabilized by regular seven‐membered hydrogen bonds within AOC* residues (Figures 6c,d), matching the conformations previously predicted by Hofmann and coworkers [42]. The 7‐helix exhibits a pitch of ∼9.0 Å with only two residues per turn, resulting in a distinct spatial arrangement of functional groups. A comparison of the torsion angles of the heterocyclic γ‐peptides is provided in Table 1. FT‐IR experiments validated the distinct H‐bonding patterns between ATC* and AOC* oligomers. AOC* oligomers **2a–f** exhibited ν(NH) at ∼3402 and 3296 cm^−1^ and ν(CO) at ∼1679 and 1650 cm^−1^ for free and H‐bonded amides, respectively (Figure 6e). Deconvolution confirmed a strong predominance of H‐bonded amide groups (Figures S22–S27; Table S37), in agreement with the 7‐Helix NMR structures. Compared to ATC* oligomers, the lower ν(NH) frequencies of the engaged amides in AOC* oligomers (∼20 cm^−1^) suggest stronger *C*_7_ hydrogen bonds, in agreement with NMR H/D experiments (Figure S14) and NBO analyses (Table S4). The CD signature of the AOC* left‐handed 7‐helix also displays a strong negative maximum but shifted to around 250 nm compared to ATC* oligomers (Figure 6f). A slight red shift with increasing oligomer length is observed, from 245 to 249 nm, together with a marked increase in signal intensity, reaching ∼72 500 deg·cm^2^·dmol^−^^1^ for the AOC* dodecamer **2f**.

**TABLE 1:** Average ϕ, θ, ζ, and ψ dihedral angles (°) of the heterocyclic γ‐amino acids in the ATC 9‐Helix, ATC* extended/7‐Helix and AOC* 7‐Helix. Torsion angles for the discontinuous ATC* 7‐helix were measured from the optimized MicroED structure of **1d**. Values for the AOC* 7‐helix correspond to averages over the 20 lowest‐energy NMR structures of oligomers **2a–c**.

Oligomer structures		*ϕ*	*θ*	*ζ*	*ψ*
**ATC 9‐Helix**	*C*_9_	−78 ± 3	127 ± 14	0 ± 3	−41 ± 4
ATC* Ext/7‐Helix	Extended	−89 ± 1	162 ± 1	−2 ± 1	−177 ± 1
	*C*_7_	−114 ± 1	−54 ± 1	0 ± 1	−178 ± 1
AOC* 7‐Helix	*C*_7_	−147 ± 7	−50 ± 5	1 ± 4	161 ± 7

## 3 Conclusion

The rational tuning of the heterocycle‐carboxamide stereoelectronic effects within heterocyclic γ‐amino acids provides a powerful strategy to control azole γ‐peptide folding and expand their conformational landscape. A simple S/N heteroatom permutation within the thiazole ring converts the canonical ATC‐based right‐handed 9‐Helix into a stretched left‐handed helix driven by alternating seven‐membered pseudoring hydrogen bonds and S···N chalcogen interactions in the solid‐state, albeit with conformational heterogeneity in solution. Removing these competing effects through a subtle S→O substitution yields oxazole‐based oligomers that adopt a stable 7‐Helix stabilized by a continuous hydrogen‐bond network. While the ATC‐based 9‐helix is reminiscent of the naturally occurring 3_10_‐helix, the geometry of 7‐helix differs markedly from any canonical protein secondary structures. Such features are expected to enable alternative interaction patterns, with biological properties to be compared to those previously explored for the 9‐Helix, including antimicrobial activity, inhibition of amyloid aggregation, and receptor‐mediated uptake. In addition, the unique topology of the 7‐helix may offer promising opportunities in enamine catalysis, which will be investigated in future studies.

## Author Contributions

**Samantha Chaise**: investigation, writing – original draft, data curation, validation, software, methodology, and visualization. **Claude Didierjean**: investigation, writing – original draft, writing – review and editing, methodology, validation, visualization, and software. **Audrey Gacogne**: investigation, writing – original draft. **Maxime Fillaudeau**: writing – original draft, investigation. **Young Kee Kang**: investigation, writing – original draft, methodology, validation, visualization, software, writing – review and editing. **Aurélien Lebrun**: data curation, software, investigation, and methodology. **Jean‐Louis Bantignies**: investigation, writing – original draft. **Dominique Housset**: investigation, writing – original draft. **Muriel Amblard**: investigation, writing – original draft. **Ludovic T. Maillard**: writing – review and editing, writing – original draft, investigation, conceptualization, funding acquisition, validation, methodology, formal analysis, project administration, resources, supervision, data curation, visualization, and software. **Baptiste Legrand**: conceptualization, investigation, funding acquisition, writing – original draft, methodology, validation, visualization, writing – review and editing, software, formal analysis, project administration, data curation, supervision, and software.

## Conflicts of Interest

The authors declare no conflicts of interest.

## Supporting information




**Supporting File**: anie73645‐sup‐0001‐SuppMat.docx.

## Data Availability

The data that support the findings of this study are available from the corresponding author upon reasonable request.
