# Supplementary material for: Azole γ‐Peptides Helix Switching via Heterocycle Substitutions
Source: Angew Chem Int Ed Engl. 2026 Jul 16;65(36):e9299244. doi: 10.1002/anie.9299244 (PMC13528534; doi:10.1002/anie.9299244)
Supplement: Supplementary file 1 [file ANIE-65-e9299244-s001.docx]

Azole γ-Peptides Helix Switching via Heterocycle Substitutions

Samantha Chaise, Claude Didierjean, Audrey Gacogne, Maxime Fillaudeau, Young Kee Kang, Aurélien Lebrun, Jean-Louis Bantignies, Dominique Housset, Muriel Amblard, Ludovic T. Maillard*, and Baptiste Legrand*

[I.	General experimental conditions	2](#_Toc229066768)

[II.	Chemical synthesis and product descriptions	2](#_Toc229066769)

[III.	Micro-ED structure determination	8](#_Toc229066770)

[IV.	Computational methods	12](#_Toc229066771)

[a.	Electrostatic and chalcogen interaction energies in ATC* and AOC* monomers.	12](#_Toc229066772)

[b.	Conformational preferences of ATC* and AOC* monomers.	13](#_Toc229066773)

[c.	CH···π interactions within ATC* oligomer **1d**.	15](#_Toc229066774)

[d.	NCIplot analyses.	15](#_Toc229066775)

[V.	NMR and molecular modelling	17](#_Toc229066776)

[a.	NMR experiments	17](#_Toc229066777)

[b.	Structure calculations	17](#_Toc229066778)

[c.	NH/ND exchange NMR experiments.	25](#_Toc229066779)

[VI.	FTIR studies	27](#_Toc229066780)

[VII.	Circular dichroism	34](#_Toc229066781)

[VIII.	2D NMR spectra of **1a-b**, **1g** and **2a-c**.	35](#_Toc229066782)

[IX.	MS-spectra	77](#_Toc229066783)

# I. General experimental conditions

Commercially available reagents and solvents were used without any further purification. Solid-phase synthesis was performed with a AmphiSpheres™ 40 RAM, 75-150 µm resin loaded at 0.31 mmol/g using Fmoc / tBu chemistry. Reactions were monitored by HPLC using an analytical Chromolith® HighResolution RP-18e column (50 x 4.6 mm, 5 μm) using a flow rate of 5.0 ml/min, and gradients from 100/0 to 0/100 eluents A/B over 5 minutes, in which eluents A = H_2_O / TFA 0.1% and B = CH_3_CN / TFA 0.1%. Detection was done at 214 nm using a Photodiode Array Detector. Retention times were reported as follows: LC: rt = min. LC-MS spectra (ESI) were recorded on an HPLC using an analytical HSS T3pept100A 1.8microns 50x2.1m using a flow rate of 0.8 mL/min, and gradients of 100/0 to 0/100 eluents A/B over 5 minutes, in which eluent solvent A = H_2_O/HCOOH 0.1% and solvent B = CH_3_CN/HCOOH 0.1%. ^1^H and ^13^C NMR spectra were recorded at room temperature in deuterated solvents. Chemical shifts (δ) are given, in this document, in parts per million, relative to TMS or relative to the solvent [^1^H NMR (600 MHz, CDCl_3_): δ ppm 7.26 ppm*,* ^13^C NMR (400 MHz, CDCl_3_): δ ppm 77.2 ppm]. The following abbreviations are used to designate the signal multiplicities: s (singlet), d (doublet), dd (doublet doublet), t (triplet), q (quartet), m (multiplet), br (broad).

# II. Chemical synthesis and product descriptions

N-Fmoc protected ATC, ATC* and AOC* were synthesized as previously described.[1][2] Solid-phase peptide synthesis (SPPS) was performed from a single batch of AmphiSpheres™ Rink Amide resin (0.50 mmol, 1.61 g, loading: 0.31 mmol/g) using Fmoc chemistry. The resin was swollen in DMF for 30 min and filtered. Each elongation cycle consisted of: (i) Fmoc deprotection was carried out with piperidine/DMF (2:8, v/v, 3 × 15 min, rt); (ii) washing (3×DMF, 3×MeOH, 3×DCM, 1×DMF, 1×MeOH, 1×DCM); (iii) coupling of Fmoc-ATC*-OH (or Fmoc-AOC*-OH; 1.75 equiv.), DIC (1.75 equiv.) and Oxyma Pure (1.75 equiv.) in NMP (30 mL/g, overnight, rt); (iv) the same washing sequence; and (v) a capping step with Ac₂O/DCM (1:1, v/v, 2 × 10 min, rt). This capping was carried out while the growing chain was still Fmoc-protected, so that only unreacted amino groups arising from incomplete couplings (truncation/deletion sequences) were acetylated, leaving the main sequence available for further elongation. Each Fmoc removal and coupling step was monitored by Kaiser test.

Couplings were carried out one residue at a time. After every second coupling, i.e. at chain lengths of 2, 4, 6, 8, 10 and 12 residues, one-sixth of the resin batch (ca. 0.083 mmol, based on the starting loading) was withdrawn, while the remaining resin was re-engaged in the next elongation cycle. Six equal fractions were thus obtained across the synthesis, affording the dimer, tetramer, hexamer, octamer, decamer and dodecamer from a single resin batch.

Each withdrawn fraction was processed as follows. The *N*-terminal Fmoc group was first removed (piperidine/DMF, 2:8, v/v, 3 × 15 min, rt) and the resin washed (3 × DMF, 3 × MeOH, 3 × DCM, 1 × DMF, 1 × MeOH, 1 × DCM); the resulting free N-terminus was then acetylated with Ac₂O/DCM (1:1, v/v, 2 × 5 min, rt) to give the *N*-acetylated, *C*-terminal carboxamide oligomer. The oligomer was cleaved from the resin with TFA/TIS/H₂O (95:2.5:2.5, v/v/v) in two successive treatments (3 h, then overnight, rt); the resin was washed (3 × DCM), and the filtrates from both treatments were combined and concentrated under reduced pressure.

Each oligomer was purified by preparative RP-HPLC on a Gilson system equipped with a C18 Luna column (100 × 21.2 mm, 5 µm; flow rate 20 mL/min; UV detection at 214 nm), using a linear gradient of B in A [A = H₂O (0.1% TFA), B = MeCN (0.1% TFA).

| **Compound** | **Gradient** | **Compound** | **Gradient** |
| --- | --- | --- | --- |
| **1a** | 30 → 50% B over 20 min | **2a** | 30 → 50% B over 20 min |
| **1b** | 45 → 70% B over 20 min | **2b** | 40 → 60% B over 20 min |
| **1c** | 55 → 75% B over 20 min | **2c** | 55 → 75% B over 20 min |
| **1d** | 75 → 90% B over 20 min | **2d** | 60 → 80% B over 20 min |
| **1e** | 75 → 90% B over 20 min | **2e** | 65 → 85% B over 20 min |
| **1f** | 75 → 90% B over 20 min | **2f** | 65 → 85% B over 20 min |
| **1g** | 75 → 90% B over 20 min |  |  |

[1] L. Mathieu, C. Bonnel, N. Masurier, L. T. Maillard, J. Martinez, V. Lisowski, “Cross-Claisen Condensation of N-Fmoc-Amino Acids - A Short Route to Heterocyclic gamma-Amino Acids” Eur. J. Org. Chem. **2015**, 2015, 2262–2270.

[2] S. Chaise, A. Gacogne, I. Garcin, R. M. de Figueiredo, J.-M. Campagne, B. Legrand, L. T. T. Maillard, “Expanding the Scope of Azole-Based γ-Amino Acids” *Synthesis* **2025**, *27* (31), 8.

Compound **1a**:

White solid, m= 29 mg, corresponding to 73% isolated yield. LC-MS (ESI+): rt = 2.73 min. m/z (%) = 472.3 (100) [M + H]^+^, 494.3 (40) [M + Na]^+^. Purity (HPLC, 214 nm) > 98%.

Compound **1b**:

White solid, m= 35 mg, corresponding to 48 %. LC-MS (ESI+): rt = 3.86 min. m/z (%) = 884.3 (40) [M + H]^+^, 906.3 (15) [M + Na]^+^. Purity (HPLC, 214 nm) > 98%.

Compound **1c**:

White solid, m= 45 mg, corresponding to 42% isolated yield. LC-MS (ESI+): rt = 4.56 min. m/z (%) = 432.9 (20) [M + 3H]^+^, 648.8 (100) [M + 2H]^+^, 1296.3 (10) [M + H]^+^, 1318.3 (5) [M + Na]^+^. Purity (HPLC, 214 nm) > 98%.

Compound **1d**:

White solid, m= 52 mg, corresponding to 37% isolated yield. LC-MS (ESI+): rt = 5.02 min. m/z (%) = 428.0 (5) [M + 4H]^+^, 570.4 (100) [M + 3H]^+^, 855.3 (70) [M + 2H]^+^. Purity (HPLC, 214 nm) > 98%.

Compound **1e**:

White solid, m= 124 mg, corresponding to 70% isolated yield. LC-MS (ESI+): rt = 5.33 min. m/z (%) = 531.5 (30) [M + 4H]^+^, 708.0 (100) [M + 3H]^+^, 1061.7 (40) [M + 2H]^+^. Purity (HPLC, 214 nm) > 98%.

Compound **1f** :

White solid, m= 55 mg, corresponding to 26% isolated yield. LC-MS (ESI+): rt = 5.56 min. m/z (%) = 507.8 (5) [M + 5H]^+^ 634.6 (100) [M + 4H]^+^, 846.0 (90) [M + 3H]^+^, 1268.3 (200) [M + 2H]^+^. Purity (HPLC, 214 nm) = 82%.

Compound **1g**:

White solid, m= 50 mg, corresponding to 58% isolated yield. LC-MS (ESI+): rt = 4.45 min. m/z (%) = 518.7 (100) [M + 2H]^+^, 1036.1 (60) [M + H]^+^, 1058.1 (30) [M + Na]^+^. Purity (HPLC, 214 nm) > 98%.

Compound **2a**:

White solid, m= 25 mg, corresponding to 68% isolated yield. LC-MS (ESI+): rt = 2.41 min. m/z (%) = 440.3 (100) [M + H]^+^, 462.3 (70) [M + Na]^+^. Purity (HPLC, 214 nm) > 98%.

Compound **2b**:

White solid, m= 31 mg, corresponding to 45% isolated yield. LC-MS (ESI+): rt = 3.33 min. m/z (%) = 410.8 (100) [M + 2H]^+^, 820.5 (40) [M + H]^+^, 842.5 (20) [M + Na]^+^. Purity (HPLC, 214 nm) > 98%.

Compound **2c** :

White solid, m= 39 mg, corresponding to 39% isolated yield. LC-MS (ESI+): rt = 3.92 min. m/z (%) = 600.9 (100) [M + 2H]^+^, 1200.8 (5) [M + H]^+^, 1222.8 (5) [M + Na]^+^. Purity (HPLC, 214 nm) > 98%.

Compound **2d** :

White solid, m= 58 mg, corresponding to 44% isolated yield. LC-MS (ESI+): rt = 4.34 min.m/z (%) = 527.9 (40) [M + 3H]^+^, 791.3 (100) [M + 2H]^+^. Purity (HPLC, 214 nm) > 98%.

Compound **2e**:

White solid, m= 44 mg, corresponding to 27% isolated yield. LC-MS (ESI+): rt = 4.64 min. m/z (%) = 654.7 (100) [M + 3H]^+^, 981.4 (50) [M + 2H]^+^. Purity (HPLC, 214 nm) > 98%.

Compound **2f**:

White solid, m= 29 mg, corresponding to 15% isolated yield. LC-MS (ESI+): rt = 4.87 min. m/z (%) = 586.9 (10) [M + 4H]^+^, 781.5 (100) [M + 3H]^+^, 1172.0 (20) [M + 2H]^+^. Purity (HPLC, 214 nm) = 93%.

# III. Micro-ED structure determination

Microcrystals of **1d** were deposited on a 400 mesh copper EM grid covered with a Lacey carbon film and loaded on a Tecnai F20 cryo-electron microscope (FEI) operated at 200 kV and equipped with a hybrid pixel Medipix3RX 512x512 detector. Six data sets of electron diffraction data were collected at 294 K from three different microcrystals of **1d**. The microcrystals were continuously rotated on an angular wedge ranging from -55˚to 55˚with 0.1835˚per frame and an exposure time of 0.125 s/frame. The camera length was set to D = 320 mm, corresponding to calibrated camera length values of 577.32 mm (resolution at detector’s edge of 1.03Å). Data were collected with a parallel beam (C2 condenser set to 78%) with spot sizes varying from 5 to 7, depending on the data set, and corresponding to dose rates from 0.03 e.Å^-2^. s^-1^ (spot size 5) to 0.007 e.Å^-2^. s^-1^ (spot size 7). The total electron dose used for the merged data set is 6.5 e.Å^-2^. The six data-sets were processed with the XDS package [1]. The unit cell parameters were determined and refined for each data sets. The average unit cell parameters, weighted by the number of reflections used for each data sets was calculated by CELLPARM from the XDS package and used for merging the six data sets with XSCALE. The precision of the unit cell parameters was estimated from the standard deviation calculated from *a*, *b*, *c* and *β* values of the 6 individual data sets. The crystal structure was solved using SHELXD [2] and refined using SHELXL [3] in Olex GUI [4], using neutral atoms and the kinematical approximation. All hydrogen atoms were placed in calculated positions using a riding model. The crystal data and statistics for **1d** are presented in the crystallographic table below. CDCC 2506122 contains the supplementary crystallographic data for this paper. This data can be obtained free of charge from The Cambridge Crystallographic Data Centre via [www.ccdc.cam.ac.uk/structures](http://www.ccdc.cam.ac.uk/structures).

Table S1: Crystal data and structure refinement for **1d**.

Identification code	**1d**

Empirical formula	C_82_H_85_N_17_O_9_S_8_

Formula weight (g.mol^-1^)	1709.17

Temperature/K	294(2)

Crystal system	monoclinic

Space group	*P*2_1_

*a*/Å	14.40(2)

*b*/Å	11.33 (1)

*c*/Å	29.04(11)

*α*/°	90

*β*/°	102.9(1)

*γ*/°	90

Volume/Å^3^	4618(19)

*Z*	2

*ρ*_calc_/g.cm^-3^	1.229

F(000)	695.0

Probe	electrons (*λ*/Å 0.02508)

2*Θ* range for data collection/°	0.102 to 1.382

Resolution range (highest resolution shell)/Å	14.03 to 1.04 (1.07 to 1.04)

Reflections collected	52,305 (3405)

Independent reflections	4226 (289)

Completeness/%	92.5 (87.3)

*R*_int_	0.239 (2.07)

*R*_meas_	0.249 (2.16)

I/σ(I)	5.86 (1.08)

CC_1/2_	99.2 (16.7)

Data/restraints/parameters	4221/1919/1144

Goodness-of-fit on *F*^2^	1.570

Final R indexes [*I*>=2σ (*I*)]	*R*_1_ = 0.1587, *wR*_2_ = 0.3777

Final R indexes [all data]	*R*_1_ = 0.2234, *wR*_2_ = 0.4108

Largest diff. peak/hole /e.Å^-3^	0.21/-0.17

Octamer **1d** crystallizes in the *P*2₁ space group, with one oligomer in the asymmetric unit. The crystallographic screw axis runs along the *b*-axis. The helical axes of the octamers are parallel to the crystallographic *c*-axis. Because the screw axis is perpendicular to the helix axes, the helices adopt alternating orientations, pointing up and down. In the crystal, the helices organize into layers composed of both up- and down-oriented helices (Figure S1A), rather than forming separate up and down layers. These layers are held together exclusively by van der Waals interactions.

Within each layer, the helices adopt an ABAB packing arrangement, where A corresponds to up-oriented helices and B to down-oriented helices (Figure S1B). Each helix is tightly packed with four neighboring helices of opposite orientation, with which it engages in multiple intermolecular interactions (Table S2, Figure S1C and S1D). The alternating conformations of even- and odd-numbered residues give rise to a helical structure with three major grooves, which are occupied by symmetry-related benzyl side chains. Each octamer is maintained by eight N–H^…^O hydrogen bonds, four C–H^…^O hydrogen bonds, and six C–H···π interactions. The latter are particularly noteworthy, as they occur between the terminal C–H groups of phenyl residues and the thiazole rings (Figure S1E and S1F). These interactions stabilize the benzyl side chains within the grooves of the helices, thereby enhancing crystal packing.

groove

groove

groove

Figure S1. Packing analysis of the crystal structure of octamer 1d. A) View of two layers of octamers in the (*b*,*c*) plane. For clarity, octamers are shown as dashed tubes, with up-oriented helices in cyan and down-oriented helices in green. The distinction between up- and down-oriented helices is relative and is mainly used to differentiate helices oriented in opposite directions. B) View of the ABAB-type arrangement in the (*a*,*b*) plane, where A corresponds to up-oriented helices and B to down-oriented helices. C) View of octamer 1d in stick representation, oriented to highlight the helical grooves. Atoms involved in intermolecular interactions in the crystal are depicted as spheres. The hydrogen atoms are omitted for clarity. The atom color code is as follows: carbon in cyan, nitrogen in blue, oxygen in red and sulfur in yellow. D) Stereo view of an up-oriented octamer surrounded by its four nearest neighbors, which are down-oriented helices. Selected benzyl side chains are shown in stick representation. The stereo view highlights the filling of the helical grooves by benzyl rings. E) Selected N-H^…^O and C-H^…^O hydrogen bonds between an up-oriented helix and a down-oriented helix. Hydrogen bonds are highlighted as black dashed lines. F) Selected C-H^…^π interaction between a benzyl side chain of an up-oriented-helix and the thiazole ring of a down oriented helix. The center of the thiazole ring is depicted as a sphere, and the C-H^…^π interaction is shown as a black dashed line.

Table S2. Intermolecular contacts in the crystal structure of octamer **1d**.

| **N–H..O and C–H..O hydrogen bonds^a^** | | | | | | |
| --- | --- | --- | --- | --- | --- | --- |
| D-H | d(D-H) (Å) | d(H..A) (Å) | <DHA (°) | d(D..A) (Å) | A sym | Sym. code |
| N-H_ATC*3 | 1.010 | 2.218 | 160.29 | 3.187 | O_ATC*7 | [-x, y-1/2, -z+1] |
| CG1-HG1C_ ATC*3 | 1.060 | 2.487 | 148.94 | 3.439 | O_ATC*7 | [-x, y-1/2, -z+1] |
| N-H_ ATC*5 | 1.010 | 2.131 | 161.14 | 3.104 | O_ATC*5 | [-x+1, y+1/2, -z+1] |
| CG1-HG1C_ATC*5 | 1.060 | 2.438 | 143.35 | 3.349 | O_ATC*5 | [-x+1, y+1/2, -z+1] |
| N-H_ATC*7 | 1.010 | 2.209 | 155.06 | 3.154 | O_ATC*3 | [-x, y-1/2, -z+1] |
| N90_H90B_Cter | 1.010 | 2.089 | 151.88 | 3.017 | O_ATC*1 | [-x+1, y+1/2, -z+1] |
| **C–H···π interactions^b^** | | | | | | |
| D-H | d(D-H) (Å) | d(H..A) (Å) | <DHA (°) | d(D..A) (Å) | Ring sym | Sym. card |
| CG5-HG5_ATC*2 | 1.1 | 2.8 | 137 | 3.6 | ATC*7 | [-x+1, y-1/2, -z+1] |
| CG5-HG5_ATC*4 | 1.1 | 2.8 | 135 | 3.6 | ATC*5 | [-x, y+1/2, -z+1] |
| CG5-HG5_ATC*6 | 1.1 | 2.9 | 132 | 3.7 | ATC*3 | [-x+1, y-1/2, -z+1] |

(**a**) Hydrogen-bond geometries were calculated using the HTAB instruction in SHELXL[5] (**b**) C–H···π interaction geometries were calculated using PyMOL (Schrödinger, LLC).

**References:**

[1] W. Kabsch, XDS, Acta Crystallographica Section D: Biological Crystallography 66(2) (2010) 125-132

[2] G.M. Sheldrick, A short history of SHELX, Acta Crystallogr A 64(Pt 1) (2008) 112-22.https://doi.org/10.1107/S0108767307043930.

[3] G.M. Sheldrick, SHELXT - integrated space-group and crystal-structure determination, Acta crystallographica. Section A, Foundations and advances 71(Pt 1) (2015) 3-8.https://doi.org/10.1107/S2053273314026370.

[4] O.V. Dolomanov, L.J. Bourhis, R.J. Gildea, J.A.K. Howard, H. Puschmann, OLEX2: a complete structure solution, refinement and analysis program, Journal of Applied Crystallography 42(2) (2009) 339-341.https://doi.org/10.1107/S0021889808042726.

[5] G.M. Sheldrick, Crystal structure refinement with SHELXL, Acta Crystallogr C Struct Chem. (2015) 71(Pt 1):3-8. https://doi.org/10.1107/S2053229614024218.

# IV. Computational methods

All quantum-mechanical calculations were performed using the Gaussian 09 program [1]. GaussView 6.0 [2] was used to visualize and to edit peptide structures. DFT calculations were carried out using the M06-2X functional method [3]. The M06-2X is a hybrid-meta-GGA functional with improved medium-range correlation energy without any corrections for long-range dispersion interactions. It has successfully predicted noncovalent interactions of small molecules and structures, as well as the relative stabilities of biological compounds such as peptides and nucleobases [4].

## a. Electrostatic and chalcogen interaction energies in ATC* and AOC* monomers.

Natural atomic charges were calculated using natural bond orbital (NBO) analysis [5] at the M06-2X/def2-TZVP level of theory (Figure S1). Key electrostatic interactions energies are reported in Table S2. Additionally, NBO analysis at the M06-2X/def2-TZVP level of theory estimated the intra-residue chalcogen interaction between the thiazole sulfur and the amide nitrogen 5 (Table S3). The strengths of hydrogen bonds and chalocogen interactions in local minima were evaluated in terms of the second-order perturbation energy (Δ*E*_2_) of the lone pair with the corresponding antibonding orbital using NBO analysis at the M06-2X/def2-TZVP level of theory. Electrostatic potentials (ESPs) were computed at the M06-2X/def2-TZVP level of theory and ESP surfaces were visualized using GaussView (Figure 3b and 4b).

Figure S2: Atomic numbering, key natural atomic charges (in e.c.u.), and hydrogen bonding distances (in Å) for extended and *C*_7_ conformations for ATC* and AOC* monomers at the M06-2X/def2-TZVP//PCM M06-2X/6-31+G(d) level of theory in chloroform.

Table S3: Electrostatic interaction energies (in kcal mol^−1^) between adjacent heteroatoms of ATC* and AOC* residues^a^.

| Ac-X-NHMe | Extended conformation | | | *C*_7_ conformation | | |
| --- | --- | --- | --- | --- | --- | --- |
|  | Atomic pair | *d* (Å) | Δ*E*_es_ | Atomic pair | *d* (Å) | Δ*E*_es_ |
| X = ATC* | N13···S6 | 2.99 | -6.1 | N13···O4 | 2.80 | +11.1 |
|  | O15···S6 | 3.63 | -5.0 | O15···O4 | 4.94 | +6.3 |
|  |  | sum | -11.1 |  | sum | +17.4 |
| X = AOC* | N13···O6 | 2.74 | +6.5 | N13···O4 | 2.83 | +10.5 |
|  | O15···O6 | 3.26 | +5.6 | O15···O4 | 5.09 | +6.1 |
|  |  | sum | +12.1 |  | sum | +16.6 |

^a^ Atomic species and numbering are defined in Figure S1. ^b^ Electrostatic energies were calculated using the equation Δ*E*_es_ (*i*, *j*) = 332 × q*_i_* q*_j_*/(ε∙*d_ij_*), where q*_i_* and q*_j_* are natural atomic charges (in e.c.u.), ε is the dielectric constant (ε = 4.71 for chloroform), and *d_ij_* is the distance between *i* and *j*.

Table S4: Second-order perturbation energies (Δ*E*_2_ in kcal mol^−1^) of the hyperconjugation between lone pair (LP) orbital and the corresponding antibonding orbital (σ***).

| Ac-X-NHMe | Conf. | Hyperconjugate pair | Δ*E*_2_ | Remark |
| --- | --- | --- | --- | --- |
| X = ATC* | Extended | LP(N13) → σ***(S6—C7) | 0.94 | Chalcogen interaction |
|  | *C*_7_ | LP(O4) → σ***(N13—H26) | 4.63 | Hydrogen bond |
| X = AOC* | Extended | LP(N13) → σ***(O6—C7) | 0.27 | Chalcogen interaction |
|  | *C*_7_ | LP(O4) → σ***(N13—H26) | 7.86 | Hydrogen bond |

## b. Conformational preferences of ATC* and AOC* monomers.

A systematic conformational search was performed using the Discovery Studio package [6] with the CHARMm force field, setting the maximum systematic conformations to 1000 and an energy threshold of 20 kcal.mol^-1^. Each backbone torsion angle (φ, θ, and ψ) was systematically varied in 60° increments. This procedure generated 66, 67 and 66, initial conformers for X = ATC, ATC* and AOC* of Ac-X-NHMe, respectively. These initial structures were first optimized at the HF/3-21G(d) level of theory, followed by subsequent optimizations at M06-2X/6-31G(d) and M06-2X/6-31+G(d) levels of theory. Further geometry optimizations were then performed in chloroform using the implicit polarizable continuum model (PCM) [7] at the M06-2X/6-31+G(d) level of theory.

For all local minima of Ac-X-NHMe with relative energies < 10 kcal mol^-1^ at the PCM M06-2X/6-31+G(d) level of theory, the relative energies (Δ*E*_s_) in chloroform were computed as the sum of the relative single-point energies (Δ*E*_0,dTZ_) obtained at the M06-2X/def2-TZVP level of theory and the relative solvation free energies (ΔΔ*G*_solv_) calculated at the PCM M06-2X/6-31+G(d) level of theory. Boltzmann populations (w) were determined from the Δ*E*_s_ values at 298 K.

Table S5: Conformational search results. Conformers, torsion angles (°), relative conformational energies (in kcal mol^−1^), and Boltzmann populations (in %) of the local minima of the Ac-X-NHMe monomer calculated at the M06-2X/def2-TZVP//PCM M06-2X/6-31+G(d) level of theory in chloroform.

| Ac-X-NHMe | Conf. | Angle φ | Angle θ | Angle ζ | Angle ψ | Δ*E*_0,dTZ_ | ΔΔ*G*_solv_ | Δ*E*_s_ | w |
| --- | --- | --- | --- | --- | --- | --- | --- | --- | --- |
| X = ATC | *C*_9_eq | -78 | 120 | -2 | -37 | 0.00 | 0.00 | 0.00 | 99.7 |
|  | *C*_7_ax | -138 | -62 | 2 | -153 | 4.98 | -0.66 | 4.32 | 0.1 |
|  | *C*_7_ax | -72 | -34 | -2 | 147 | 5.42 | -1.09 | 4.33 | 0.1 |
|  | Extended | -156 | 148 | -2 | -157 | 5.22 | -0.88 | 4.34 | 0.1 |
|  | *C*_9_ | 62 | -119 | 6 | 55 | 5.33 | -0.50 | 4.83 | 0.0 |
|  | Extended | -156 | 136 | -3 | 33 | 5.74 | -0.85 | 4.89 | 0.0 |
|  | *C*_7_eq | 56 | 51 | 2 | -157 | 6.91 | -0.39 | 6.52 | 0.0 |
| X = ATC* | *C*_7_ax | -94 | -55 | 2 | -177 | 0.00 | 0.00 | 0.00 | 79.4 |
|  | Extended | -73 | 159 | -2 | -175 | 2.78 | -1.66 | 1.11 | 12.2 |
|  | *C*_7_eq | 64 | 68 | 0 | 175 | 0.92 | 0.47 | 1.39 | 7.6 |
|  | *C*_9_eq | -72 | 124 | -2 | -48 | 6.60 | -3.62 | 2.98 | 0.5 |
|  | Extended | -145 | 73 | 1 | 177 | 4.95 | -1.65 | 3.29 | 0.3 |
|  | *C*_9_ax | 61 | -119 | 5 | 66 | 10.59 | -3.47 | 7.11 | 0.0 |
| X = AOC* | *C*_7_ax | -139 | -44 | 2 | -178 | 0.00 | 0.00 | 0.00 | 95.5 |
|  | *C*_7_eq | 58 | 58 | 1 | 179 | 4.01 | -1.56 | 2.45 | 1.5 |
|  | Extended | 74 | 158 | -3 | -179 | 6.14 | -3.64 | 2.50 | 1.4 |
|  | Extended | -65 | 158 | -1 | -176 | 2.22 | 0.37 | 2.59 | 1.2 |
|  | *C*_9_eq | -76 | 117 | -1 | -36 | 4.85 | -1.58 | 3.27 | 0.4 |
|  | Extended | -154 | 140 | -2 | -177 | 6.22 | -1.13 | 5.09 | 0.0 |
|  | *C*_9_ax | 63 | -117 | 6 | 52 | 9.64 | -3.68 | 5.96 | 0.0 |

Figure S3: Three-dimensional structures and pseudo-Newman representations of the Ac-X-NHMe local minima: *C*_9_eq, *C*_7_ax, *C*_7_eq and extended conformers.

## c. CH···π interactions within ATC* oligomer **1d**.

The ATC* octamer microED structure was first optimized at the PCM M06-2X/6-31G(d) level of theory in chloroform (Figure S3a). In the stretched helical conformation of the ATC* oligomers, the τ-methyl group attached to the thiazole ring of the *i* residue engages in a CH···π interaction with the thiazole ring of the (*i*+3) residue.

Figure S4: a) Overlay of the microED structure of the ATC* octamer **1d** (yellow) with the DFT-optimized structure computed at the PCM M06-2X/6-31G(d) level of theory in chloroform (grey). The RMSD calculated on the ATC* Cγ atoms is 1.89 Å. b) Distances between the τ-methyl group of ATC*(i) and the centroid of the thiazole ring of the i+3 residue. The average C^τ^(i)_Me_···thiazole(i+3) centroid distance is 4.1 ± 0.5 Å. c) Representative ATC* dimer optimized at the PCM M06-2X/6-31G(d) level of theory in chloroform. The interaction energy is 1.2 kcal mol^-1^, computed using the counterpoise method with basis set superposition error (BSSE) correction [8] at the M06-2X/def2-TZVP level of theory.

## d. NCIplot analyses.

Non-covalent interaction (NCI) analyses were carried out using NCIplot4.4. [9] Wavefunction files were generated from single-point DFT calculations at the M06-2X/6-31+G(d) level on optimized geometries. The electron density (ρ) and reduced density gradient (RDG) were computed on a cubic grid with default parameters. NCI isosurfaces were generated using an RDG isovalue of 0.5 a.u., and colored according to the sign of the second eigenvalue of the Hessian of ρ [sign(λ₂)ρ] from -4.00 to 4.00: blue (strong attractive), green (weak attractive/van der Waals), and red (repulsive). The resulting 3D surfaces were visualized using Pymol 3.5.1, [10] enabling identification of intramolecular contacts such as *C*_7_ hydrogen bonds, S···N electrostatic–chalcogen interactions, and long-range CH···π interactions within ATC*, ATC, and AOC* oligomers.

Figure S5: NCIplot visualization of non-covalent interactions within ATC* and AOC* in extended and C7 conformations.

Figure S6: NCIplot visualization of non-covalent interactions within ATC* octamer **1d** DFT-optimized microED structure.

**References:**

[1] M. J. Frisch, et al., Gaussian 09, Revision D.01, Gaussian, Inc., Wallingford, CT: 2013

[2] R. D. Dennington II, T. A. Keith, J. Millam, GaussView (Version 6.0), Gaussian, Inc., Wallingford, CT 2016.

[3] Y. Zhao, D. G. Truhlar, *Theor. Chem. Acc.* 2008, 120, 215.

[4] Y. Zhao, D. G. Truhlar, *Chem*. *Phys*. *Lett*. 2011, 502, 1.

[5] Weinhold, F. in Encyclopedia of Computational Chemistry, ed. Schleyer, P. v. R. ; Allinger, N. L.; Clark, T.; Gasteiger, J.; Kollman, P. A.; Schaefer, H. F., III; Schreiner, P. R.; Wiley, Chichester, 1998; vol. 3, pp. 1792-1811.

[6] BIOVIA Discovery Studio, Version 16, Dassault Systèmes Co., Waltham, Mass, USA, 2015.

[7] B. Mennucci, R. Cammi, J. Tomasi, *J. Chem. Phys.* 1999, 110, 6858.

[8] S. F. Boys, F. Bernardi, *Mol. Phys.* 1970, 19, 553.

[9] J. Contreras-García, E. R. Johnson, S. Keinan, R. Chaudret, J.-P. Piquemal, D. N. Beratan, W. Yang, J. Chem. Theory Comput. 2011, 7, 625–632. V. Polo, P. Fuentealba, *J. Chem. Theory Comput.* 2022, *18*, 7180.

[10] DeLano, W. L. *The PyMOL Molecular Graphics System*; DeLano Scientific: San Carlos, CA, 2002.

# V. NMR and molecular modelling

## a. NMR experiments

The NMR samples typically contained 2 mM of **1a-g** and **2a-f** in CDCl_3_. The spectra were recorded at various temperatures (typically 253K and 298K) on a Bruker Avance 600 AVANCE III spectrometer equipped with a 5 mm triple-resonance TCI cryoprobe Prodigy (^1^H, ^13^C, ^15^N) at the UAR 2041 “Plateforme d'Analyses et de Caractérisations” (PAC) Chimie Balard Montpellier. Homonuclear 2-D spectra COSY, TOCSY and ROESY were typically recorded in the phase-sensitive mode using the States-TPPI method as data matrices of 64-400 real (t1) × 2048 (t2) complex data points; 8-64 scans per t1 increment with 1.2-1.5 s recovery delay and spectral width of 7212 Hz in both dimensions were used. The mixing times were 120 ms for TOCSY and 200 ms for the ROESY experiments. In addition, 2D heteronuclear spectra ^13^C/^15^N-HSQC and ^13^C/^15^N-HMBC were performed (8-32 scans, 64-512 real (t1) × 2048 (t2) complex data points). Spectra were processed with Topspin (Bruker Biospin) and visualized with Topspin or NMRview [1] on a Linux station. The matrices were zero-filled to 1024 (t1) x 2048 (t2) points after apodization by shifted sine-square multiplication and linear prediction in the F1 domain. Chemical shifts were referenced to the tetramethylsilane (TMS). ^1^H, ^15^N and ^13^C chemical shifts were assigned according to classical procedures as previously described [2].

## b. Structure calculations

NOE cross-peaks were integrated and assigned within the NMRView software [1]. The volumes of NOE peaks between methylene pair protons were used as reference of 1.8 Å. The lower bound for all restraints was fixed at 1.8 Å and upper bounds at 2.7, 3.3 and 5.0 Å, for strong, medium and weak correlations, respectively. Pseudo-atoms corrections of the upper bounds were applied for unresolved aromatic, methylene and methyl protons signals as described previously [2]. Structure calculations were performed with AMBER 22 [3] in two stages: cooking, simulated annealing in vacuum. The cooking stage was performed at 1000 K to generate 100 initial random structures. Simulated annealing calculations (SA) were carried during 20 ps (20000 steps, 1 fs long) as described elsewhere. First, the temperature was risen quickly and was maintained at 1000 K for the first 5000 steps, then the system was cooled gradually from 1000 K to 100 K from step 5001 to 18000 and finally the temperature was brought to 0 K during the 2000 remaining steps. For the 3000 first steps, the force constant of the distance restraints was increased gradually from 2.0 kcal.mol^-1^.Å to 20 kcal.mol^-1^.Å. For the rest of the simulation (step 3001 to 20000), the force constant is kept at 20 kcal.mol^-1^.Å. The 15 lowest energy structures with no violations > 0.3 Å were considered as representative of the compound structure. The lowest energy structure of compounds **2a**, **2b** et **2c** were optimized at the PCM M06-2X/6-31G(d) level of theory in chloroform using Gaussian 09 [4]. The representation and quantitative analysis were carried out using MOLMOL [5], PyMOL (Delano Scientific).

Figure S7: Nomenclature used for the various atoms of each residue and dihedral angles description.

Figure S8: ^1^H NMR spectra of **1a**-**1f** in CDCl_3_ at 298K. NH amide protons of even-numbered residues are shown in cyan.

Table S6: ^1^H NMR chemical shifts in ppm for oligomer **1a** (2 mM) in CDCl_3_ at 298 K.

| Residue | HN | Hγ | Hδ | Hζ | Hη | Hθ | Hτ | Others |
| --- | --- | --- | --- | --- | --- | --- | --- | --- |
| Ac | - | - | - | - | - | - | - | CH_3_: 1.91 |
| Res. 1 | 8.19 | 5.51 | 1.43 | - | - | - | 2.63 | - |
| Res. 2 | 9.09 | 6.04 | 3.38,3.27 | 7.22 | 7.25 | 7.20 | 2.57 | - |
| NH_2_ | 7.38, 5.84 | - | - | - | - | - | - | - |

Table S7: ^13^C and ^15^N NMR chemical shifts in ppm for oligomer **1a** (2 mM) in CDCl_3_ at 298 K.

| Residue | NH | Cα | Cβ | Cγ | Cδ | Cε | Cζ | Cη | Cθ | Cτ | Others |
| --- | --- | --- | --- | --- | --- | --- | --- | --- | --- | --- | --- |
| Ac | - | - | - | - | - | - | - | - | - | - | CH_3_: 23.4  CO: 170.9 |
| Res. 1 | 131.8 | 142.0 | 146.3 | 41.5 | 22.3 | - | - | - | - | 19.0 | CO: *nd*  Cτ_1_: 161.6 |
| Res. 2 | 127.1 | 141.7 | 146.7 | 48.2 | 42.1 | 137.2 | 129.4 | 128.4 | 126.9 | 19.0 | CO: *nd*  Cτ_1_: 162.4 |
| NH_2_ | 100.5 | - | - | - | - | - | - | - | - | - | - |

*nd*: not determined

Table S8: ^1^H NMR chemical shifts (in ppm) for oligomer **1b** (2 mM) in CDCl_3_ at 298 K.

| Residue | HN | Hγ | Hδ | Hζ | Hη | Hθ | Hτ | Others |
| --- | --- | --- | --- | --- | --- | --- | --- | --- |
| Ac | - | - | - | - | - | - | - | CH_3_: 1.90 |
| Res. 1 | 8.32 | 5.46 | 1.41 | - | - | - | 2.59 | - |
| Res. 2 | 9.05 | 6.04 | 3.41, 3.33 | 7.20 | 7.16 | 7.18 | 2.54 | - |
| Res. 3 | 8.87 | 5.97 | 1.64 | - | - | - | 2.63 | - |
| Res. 4 | 9.20 | 5.99 | 3.25, 3.33 | 7.17 | 7.22 | 7.18 | 2.57 | - |
| NH_2_ | 5.78, 7.39 | - | - | - | - | - | - | - |

Table S9: ^13^C and ^15^N NMR chemical shifts (in ppm) for oligomer **1b** (2 mM) in CDCl_3_ at 298 K.

| Residue | NH | Cα | Cβ | Cγ | Cδ | Cε | Cζ | Cη | Cθ | Cτ | Others |
| --- | --- | --- | --- | --- | --- | --- | --- | --- | --- | --- | --- |
| Ac | - | - | - | - | - | - | - | - | - | - | CH_3_: 22.6  CO: 170.4 |
| Res. 1 | 131.4 | 143.5 | 147.4 | 40.6 | 21.3 | - | - | - | - | 19.0 | CO: *nd*  Cτ_1_: 162.9 |
| Res. 2 | 125.6 | 143.1 | 148.2 | 47.3 | 41.1 | 137.7 | 129.4 | 128.4 | 126.7 | 19.0 | CO: *nd*  Cτ_1_: 163.8 |
| Res. 3 | 129.6 | 143.5 | 149.3 | 42.0 | 21.8 | - | - | - | - | 19.0 | CO: *nd*  Cτ_1_: 162.9 |
| Res. 4 | 127.4 | 144.0 | 146.5 | 47.4 | 41.1 | 137.7 | 129.5 | 128.4 | 126.7 | 19.0 | CO: *nd*  Cτ_1_: 163.4 |
| NH_2_ | 100.2 | - | - | - | - | - | - | - | - | - | - |

*nd*: not determined.

Table S10: ^1^H NMR chemical shifts (in ppm) for oligomer **1g** (2 mM) in CDCl_3_ at 298 K.

| Residue | HN | Hγ | Hδ | Hζ | Hη | Hθ | Hτ | Others |
| --- | --- | --- | --- | --- | --- | --- | --- | --- |
| Ac | - | - | - | - | - | - | - | CH_3_: 1.87 |
| Res. 1 | 8.48 | 5.50 | 3.08 | 6.95 | 7.12 | 7.17 | 2.55 | - |
| Res. 2 | 9.27 | 6.02 | 3.26, 3.31 | 7.16 | 7.21 | 7.17 | 2.50 | - |
| Res. 3 | 9.14 | 6.11 | 3.38, 3.41 | 7.21 | 7.18 | 7.17 | 2.59 | - |
| Res. 4 | 9.31 | 6.02 | 3.25, 3.33 | 7.15 | 7.21 | 7.17 | 2.56 | - |
| NH_2_ | 6.00, 7.45 | - | - | - | - | - | - | - |

Table S11: ^13^C and ^15^N NMR chemical shifts (in ppm) for oligomer **1g** (2 mM) in CDCl_3_ at 298 K.

| Residue | NH | Cα | Cβ | Cγ | Cδ | Cε | Cζ | Cη | Cθ | Cτ | Others |
| --- | --- | --- | --- | --- | --- | --- | --- | --- | --- | --- | --- |
| Ac | - | - | - | - | - | - | - | - | - | - | CH_3_: 22.6  CO: 170.4 |
| Res. 1 | 129.1 | 142.5 | 143.8 | 47.3 | 41.8 | 137.6 | 129.4 | 128.4 | 126.5 | 18.9 | CO: 161.5  Cτ_1_: 162.3 |
| Res. 2 | 127.4 | 142.5 | 145.1 | 48.0 | 42.2 | 137.6 | 129.6 | 128.4 | 126.5 | 18.9 | CO: 162.1  Cτ_1_: 162.0 |
| Res. 3 | 125.9 | 141.6 | 146.8 | 48.3 | 41.8 | 137.6 | 129.4 | 128.4 | 126.5 | 18.9 | CO: 161.5  Cτ_1_: 162.0 |
| Res. 4 | 126.5 | 141.8 | 145.1 | 48.0 | 42.2 | 137.6 | 129.4 | 128.4 | 126.5 | 18.9 | CO: *nd*  Cτ_1_: 161.9 |
| NH_2_ | 101.0 | - | - | - | - | - | - | - | - | - | - |

*nd*: not determined.

Figure S9: NMR temperature-dependent behaviours of amide protons in **1b**.

Figure S10: 1H NMR spectra of **1d** at 200 µM and 2 mM.

Figure S11: Comparison of the ^1^H NMR spectra for tetramers **1b** and **1g**.

Figure S12: ^1^H NMR spectra of **2a-2f** in CDCl_3_ at 253 K.

Figure S13: 1H NMR spectra of **2c** at 200 µM and 2 mM.

Table S12: ^1^H NMR chemical shifts in ppm for oligomer **2a** (2 mM) in CDCl_3_ at 298 K.

| Residue | HN | Hγ | Hδ | Hζ | Hη | Hθ | Hτ | Others |
| --- | --- | --- | --- | --- | --- | --- | --- | --- |
| Ac | - | - | - | - | - | - | - | CH_3_: 1.97 |
| Res. 1 | 8.78 | 5.37 | 1.37 | - | - | - | 2.45 | - |
| Res. 2 | 9.16 | 5.81 | 3.20 | 7.13 | 7.23 | 7.19 | 2.34 | - |
| NH_2_ | 6.93, 5.87 | - | - | - | - | - | - | - |

Table S13: ^13^C and ^15^N NMR chemical shifts in ppm for oligomer **2a** (2 mM) in CDCl_3_ at 298 K.

| Residue | NH | Cα | Cβ | Cγ | Cδ | Cε | Cζ | Cη | Cθ | Cτ | Others |
| --- | --- | --- | --- | --- | --- | --- | --- | --- | --- | --- | --- |
| Ac | - | - | - | - | - | - | - | - | - | - | CH_3_: 23.1  CO: 169.7 |
| Res. 1 | 126.6 | 128.7 | 157.2 | 42.1 | 21.2 | - | - | - | - | 13.6 | CO: 161.3  Cτ_1_: 158.6 |
| Res. 2 | 119.2 | 129.3 | 155.6 | 46.9 | 41.2 | 136.2 | 129.3 | 128.5 | 126.8 | 13.5 | CO: *nd*  Cτ_1_: 159.0 |
| NH_2_ | 99.0 | - | - | - | - | - | - | - | - | - | - |

*nd*: not determined.

Table S14: ^1^H NMR chemical shifts in ppm for oligomer **2b** (2 mM) in CDCl_3_ at 243 K.

| Residue | HN | Hγ | Hδ | Hζ | Hη | Hθ | Hτ | Others |
| --- | --- | --- | --- | --- | --- | --- | --- | --- |
| Ac | - | - | - | - | - | - | - | CH_3_: 1.94 |
| Res. 1 | 8.87 | 5.34 | 1.34 | - | - | - | 2.48 | - |
| Res. 2 | 9.52 | 5.77 | 3.16 | 7.17 | 7.22 | 7.19 | 2.44 | - |
| Res. 3 | 9.56 | 5.76 | 1.54 | - | - | - | 2.51 | - |
| Res. 4 | 9.44 | 5.83 | 3.25, 3.29 | 7.08 | 7.23 | 7.19 | 2.31 | - |
| NH_2_ | 7.07, 5.99 | - | - | - | - | - | - | - |

*nd*: not determined.

Table S15: ^13^C and ^15^N NMR chemical shifts in ppm for oligomer **2b** (2 mM) in CDCl_3_ at 243 K.

| Residue | NH | Cα | Cβ | Cγ | Cδ | Cε | Cζ | Cη | Cθ | Cτ | Others |
| --- | --- | --- | --- | --- | --- | --- | --- | --- | --- | --- | --- |
| Ac | - | - | - | - | - | - | - | - | - | - | CH_3_: 23.5  CO: 169.8 |
| Res. 1 | 126.9 | 128.7 | 156.9 | 42.1 | 21.4 | - | - | - | - | 13.8 | CO: 161.4  Cτ_1_: 158.5 |
| Res. 2 | 118.7 | 130.3 | 154.7 | 46.9 | 41.4 | 136.6 | 129.5 | 128.5 | 126.8 | 13.8 | CO: 160.8  Cτ_1_: 159.0 |
| Res. 3 | 119.8 | 129.3 | 156.4 | 41.7 | 21.5 | - | - | - | - | 13.8 | CO: 161.3  Cτ_1_: 159.0 |
| Res. 4 | 118.6 | 129.7 | 155.6 | 47.0 | 41.1 | 136.6 | 129.3 | 128.5 | 126.8 | 13.5 | CO: *nd*  Cτ_1_: 159.0 |
| NH_2_ | 99.2 | - | - | - | - | - | - | - | - | - | - |

*nd*: not determined.

Table S16: ^1^H NMR chemical shifts in ppm for oligomer **2c** (2 mM) in CDCl_3_ at 243 K.

| Residue | HN | Hγ | Hδ | Hζ | Hη | Hθ | Hτ | Others |
| --- | --- | --- | --- | --- | --- | --- | --- | --- |
| Ac | - | - | - | - | - | - | - | CH_3_: 1.94 |
| Res. 1 | 8.89 | 5.34 | 1.34 | - | - | - | 2.50 | - |
| Res. 2 | 9.55 | 5.77 | 3.16 | 7.19 | 7.18 | 7.13 | 2.43 | - |
| Res. 3 | 9.57 | 5.75 | 1.54 | - | - | - | 2.51 | - |
| Res. 4 | 9.54 | 5.84 | 3.19, 3.27 | 7.09 | 7.18 | 7.13 | 2.35 | - |
| Res. 5 | 9.43 | 5.75 | 1.53 | - | - | - | 2.51 | - |
| Res. 6 | 9.42 | 5.83 | 3.29, 3.25 | 7.09 | 7.23 | 7.13 | 2.31 | - |
| NH_2_ | 7.08, 5.98 | - | - | - | - | - | - | - |

Table S17: ^13^C and ^15^N NMR chemical shifts in ppm for oligomer 2c (2 mM) in CDCl_3_ at 243 K.

| Residue | NH | Cα | Cβ | Cγ | Cδ | Cε | Cζ | Cη | Cθ | Cτ | Others |
| --- | --- | --- | --- | --- | --- | --- | --- | --- | --- | --- | --- |
| Ac | - | - | - | - | - | - | - | - | - | - | CH_3_: 23.6  CO: 169.8 |
| Res. 1 | 127.0 | 128.7 | 157.2 | 42.1 | 21.4 | - | - | - | - | 14.2 | CO: 161.0  Cτ1: 159.0 |
| Res. 2 | 120.1 | 130.2 | 154.7 | 47.0 | 41.5 | 136.1 | 129.5 | 128.4 | 126.7 | 14.0 | CO: 161.0  Cτ1: 159.3 |
| Res. 3 | 121.5 | 129.3 | 156.4 | 41.7 | 21.4 | - | - | - | - | 14.3 | CO: 161.0  Cτ1: 159.3 |
| Res. 4 | 122.6 | 130.3 | 155.5 | 47.0 | 41.1 | 136.4 | 129.3 | 128.5 | 126.7 | 14.1 | CO: 161.0  Cτ1: 159.3 |
| Res. 5 | 119.6 | 129.3 | 156.4 | 41.7 | 21.4 | - | - | - | - | 14.3 | CO: 161.0  Cτ1: 159.0 |
| Res. 6 | 122.2 | 129.5 | 155.5 | 47.0 | 41.1 | 136.4 | 129.3 | 128.5 | 126.7 | 13.7 | CO: *nd*  Cτ1: 159.3 |
| NH_2_ | 100.1 | - | - | - | - | - | - | - | - | - | - |

*nd*: not determined.

Table S18: ^1^H NMR chemical shifts in ppm for oligomer **2d** (2 mM) in CDCl_3_ at 253 K.

| Residue | HN | Hγ | Hδ | Hζ | Hη | Hθ | Hτ | Others |
| --- | --- | --- | --- | --- | --- | --- | --- | --- |
| Ac | - | - | - | - | - | - | - | CH_3_: 1.94 |
| Res. 1 | 8.95 | 5.34 | 1.35 | - | - | - | 2.48 | - |
| Res. 2 | 9.50 | 5.78 | 3.17 | 7.17 | 7.17 | 7.21 | 2.42 | - |
| Res. 3 | 9.51 | 5.74 | 1.54 | - | - | - | 2.51 | - |
| Res. 4 | 9.52 | 5.84 | 3.20, 3.27 | 7.08 | 7.08 | 7.08 | 2.33 | - |
| Res. 5 | 9.40 | 5.73 | 1.53 | - | - | - | 2.51 | - |
| Res. 6 | 9.47 | 5.85 | 3.20, 3.26 | 7.08 | 7.08 | 7.08 | 2.33 | - |
| Res. 7 | 9.32 | 5.76 | 1.53 | - | - | - | 2.50 | - |
| Res. 8 | 9.36 | 5.84 | 3.24, 3.28 | 7.08 | 7.16 | 7.12 | 2.29 | - |
| NH_2_ | 7.08, 6.08 | - | - | - | - | - | - | - |

Table S19: ^13^C and ^15^N NMR chemical shifts in ppm for oligomer **2d** (2 mM) in CDCl_3_ at 253 K.

| Residue | NH | Cα | Cβ | Cγ | Cδ | Cε | Cζ | Cη | Cθ | Cτ | Others |
| --- | --- | --- | --- | --- | --- | --- | --- | --- | --- | --- | --- |
| Ac | - | - | - | - | - | - | - | - | - | - | CH_3_: 23.4  CO: 169.9 |
| Res. 1 | 127.4 | 128.8 | 157.0 | 42.0 | 21.4 | - | - | - | - | 14.1 | CO: 160.9  Cτ1: 158.9 |
| Res. 2 | 120.0 | 130.1 | 154.7 | 47.1 | 41.4 | 136.6 | 129.4 | 128.4 | 126.8 | 14.1 | CO: 160.9  Cτ1: 159.0 |
| Res. 3 | 120.0 | 129.2 | 156.3 | 41.7 | 21.2 | - | - | - | - | 14.2 | CO: 160.9  Cτ1: 159.0 |
| Res. 4 | 122.7 | 130.1 | 155.6 | 46.9 | 41.0 | 136.8 | 129.3 | 128.4 | 126.8 | 14.0 | CO: 160.9  Cτ1: 159.0 |
| Res. 5 | 122.4 | 129.2 | 156.3 | 41.7 | 21.2 | - | - | - | - | 14.2 | CO: 160.9  Cτ1: 159.0 |
| Res. 6 | 120.0 | 130.1 | 155.2 | 46.9 | 41.0 | 136.8 | 129.3 | 128.4 | 126.8 | 14.0 | CO: 160.9  Cτ1: 159.0 |
| Res. 7 | 122.1 | 129.2 | 156.3 | 41.7 | 21.2 | - | - | - | - | 14.2 | CO: 160.9  Cτ1: 158.9 |
| Res. 8 | 119.5 | 130.1 | 155.6 | 46.9 | 41.0 | 136.8 | 129.3 | 128.4 | 126.8 | 13.8 | CO: *nd*  Cτ1: 159.0 |
| NH_2_ | 100.6 | - | - | - | - | - | - | - | - | - | - |

*nd*: not determined.

Table S20: Coupling constants values ^3^*J*(HN,Hγ) for **1a-b** and **2a-d** (in Hz).

| Residue | 1a | 1b | 1g | 2a | 2b | 2c | 2d |
| --- | --- | --- | --- | --- | --- | --- | --- |
| Res. 1 | 9.3 | 8.9 | 9.3 | 9.0 | 9.1 | 9.1 | 9.1 |
| Res. 2 | 6.8 | 7.8 | 7.9 | 9.6 | 9.6 | o | 9.5 |
| Res. 3 | - | 7.8 | 8.2 | - | 9.3 | o | 9.2 |
| Res. 4 | - | b | 7.9 | - | 9.6 | o | 9.3 |
| Res. 5 | - | - | - | - | - | 9.5 | 9.2 |
| Res. 6 | - | - | - | - | - | 9.3 | 9.5 |
| Res. 7 | - | - | - | - | - | - | 9.1 |
| Res. 8 | - | - | - | - | - | - | 9.6 |

*b*: broad; o: overlapped

Table S21: Inter-residue NOEs unambiguously assigned in the ROESY spectra of in CDCl_3_ at 243 K. Strong < 2.7 Å < medium < 3.3 Å < weak < 5.0 Å. NOEs characteristic of the 7-helix are shown in bold.

| NOE correlations | 2a | 2b | 2c |
| --- | --- | --- | --- |
| 1.HN-Ac | Weak | Weak | Weak |
| 1.Hγ-Ac | Weak | Weak | Weak |
| 1.HN-1.Hγ | Weak | Weak | Weak |
| 1.HN-1.Hδ | Weak | Weak | Weak |
| 1.HN-2.Hγ | Weak | Weak | Weak |
| 1.HN-2.Hδ | Weak | Weak | Weak |
| 1.Hτ-3.Hγ | - | Weak | o |
| 1.Hτ-4.Hγ | - | Weak | o |
| 2.HN-2.Hγ | Weak | Weak | Weak |
| 2.HN-2.Hδ | Weak | Weak | Weak |
| 2.HN-2.Hζ | Weak | Weak | Weak |
| 2.HN-1.Hτ | Weak | Weak | Weak |
| 2.Hτ-NH_2_ | Weak | - | - |
| 2.Hδ-NH_2_ | Weak | - | - |
| 2.HN-3.Hδ | - | Weak | o |
| 2.HN-3.Hγ | - | Weak | o |
| 3.HN-3.Hγ | - | Weak | Weak |
| 3.HN-3.Hδ | - | Weak | Weak |
| 3.HN-4.Hγ | - | Weak | Weak |
| 3.HN-4.Hδ | - | Weak | Weak |
| 4.HN-4.Hγ | - | Weak | Weak |
| 4.HN-4.Hδ | - | Weak | Weak |
| 4.HN-4.Hζ | - | Weak | Weak |
| 4.HN-3.Hτ | - | Weak | Weak |
| 4.Hτ-NH_2_ | - | Weak | - |
| 4.Hδ-NH_2_ | - | Weak | - |
| 4.HN-5.Hδ | - | - | Weak |
| 4.HN-5.Hγ | - | - | o |
| 5.HN-5.Hγ | - | - | Weak |
| 5.HN-5.Hδ | - | - | Weak |
| 5.HN-6.Hγ | - | - | Weak |
| 5.HN-6.Hδ | - | - | o |
| 5.HN-4.Hτ | - | - | Weak |
| 6.HN-6.Hγ | - | - | Weak |
| 6.HN-6.Hδ | - | - | Weak |
| 6.HN-6.Hζ | - | - | Weak |
| 6.HN-5.Hτ | - | - | Weak |
| 6.Hτ-NH_2_ | - | - | Weak |
| 6.Hδ-NH_2_ | - | - | o |

*nd*: not detected; *o*: overlapped

Table S22: Root Mean Square Deviations (RMSD, in Å) of the 20 lowest energy NMR structures in CDCl_3_ at 243 K.

| Atom selection | 2a | 2b | 2c |
| --- | --- | --- | --- |
| all atoms | 0.840 | 1.395 | 1.438 |
| all heavy atoms | 0.457 | 1.113 | 1.157 |
| backbone heavy atoms | 0.053 | 0.646 | 0.734 |

Table S23: Backbone dihedral angles (°) in CDCl_3_ (average values on the 20 lowest energy structures).

|  | Angles | 2a | 2b | 2c |
| --- | --- | --- | --- | --- |
| Res. 1 | φ | -140.6 ± 1.5° | -82.2 ± 0.7° | -116.1 ± 26.4° |
|  | θ | -40.7 ± 1.2° | -46.3 ± 1.0° | -47.8 ± 1.02 |
|  | ζ | 0.1 ± 0.8° | 0.4 ± 0.8° | 0.7 ± 0.7° |
|  | ψ | 177.3 ± 1.6° | 170.3 ± 2.5° | -172.3 ± 2.1° |
| Res. 2 | φ | -134.8 ± 1.0° | -151.6 ± 1.0° | -131.8 ± 6.0° |
|  | θ | -45.2 ± 4.5° | -47.0 ± 1.9° | -46.4 ± 3.7° |
|  | ζ | -3.7 ± 3.8° | -1.3 ± 1.3° | -1.2 ± 0.8° |
|  | ψ | -164.9 ± 0.8° | 174.0 ± 1.3° | 175.6 ± 3.7° |
| Res. 3 | φ | - | -142.3 ± 4.7° | -135.0 ± 7.0° |
|  | θ | - | -53.7 ± 1.4° | -47.2 ± 1.4° |
|  | ζ | - | 3.6 ± 1.4° | -1.4 ± 0.9° |
|  | ψ | - | -166.4 ± 3.7° | 176.2 ± 2.9° |
| Res. 4 | φ | - | -145.2 ± 4.3° | -146.6 ± 3.4° |
|  | θ | - | 18.4 ± 110.5° | -54.2 ± 8.2° |
|  | ζ | - | -0.8 ± 0.8° | 2.6 ± 1.1° |
|  | ψ | - | -172.7 ± 1.1° | -165.2 ± 5.0° |
| Res. 5 | φ | - | - | -89.4 ± 14.7° |
|  | θ | - | - | -46.7 ± 1.4° |
|  | ζ | - | - | -2.4 ± 1.0° |
|  | ψ | - | - | 175.0 ± 2.9° |
| Res. 6 | φ | - | - | -145.0 ± 4.3° |
|  | θ | - | - | -32.1 ± 67.6° |
|  | ζ | - | - | 1.3 ± 1.1° |
|  | ψ | - | - | -174.8 ± 1.3° |

## c. NH/ND exchange NMR experiments.

Amide H/D exchange NMR experiments were conducted to assess the solvent accessibility and conformational dynamics of amide protons within the oligomers. Exchange kinetics primarily reflect the accessibility of amide protons to the solvent. Thus, slow exchange rates are characteristic of protected sites, which may result from hydrogen bonding networks or limited solvent access due to structural burial, whereas rapidly exchanging amide protons typically correspond to solvent-exposed protons.

NMR experiments were performed at 293 K on compounds **1d** and **2c** in CDCl_3_ at a concentration of 1 mM. CD₃OD (5% v/v) was added directly to the NMR tube, and the solution was briefly mixed prior to data acquisition. The exchange process was monitored by ¹H NMR, with spectra recorded after 5, 10, 20, 30, 60, 120, 180, and 240 min from the time of CD_3_OD addition. Signal intensities were monitored over time to follow the disappearance of amide proton resonances. The decay of amide proton resonances was fitted using a mono-exponential function I(t)=I_0_ e^−k^_ex_^t^ + c, to extract apparent exchange rate constants (k_ex_). Resonances affected by spectral overlap could not be reliably integrated and were therefore excluded from the kinetic analysis.

Figure S14: NH/ND exchange of oligomers **1b** and **2c**.

Based on MicroED and NMR structures, oligomers **1d** and **2c** adopt 7-helical structures stabilized by discontinuous and regular hydrogen bonding networks, respectively. As expected, for both **1d** and **2c**, the highly solvent-exposed amide proton of the N-terminal residue rapidly disappeared, displaying short half-lives of 22 min (k ≈ 0.031 min^-1^) and 7 min (k ≈ 0.09 min^-1^), respectively. Overall, within each oligomer, the other amide protons display similar exchange kinetics, whereas distinct kinetic regimes are observed between **1d** and **2c**. The exchange rates of several amide protons could not be reliably determined due to signal overlap. Quantitative analysis was therefore performed on odd-numbered amide resonances of **1d** in the 8.7–9.0 ppm region (named a, b and c in Figure S14), as well as on the amide protons of residues 5 and 6 in oligomer **2c**. The former display half-lives of approximately 80 min (k ≈ 8.7 × 10^-3^ min^-1^), consistent with moderately protected sites. In compound **2c**, residues 5 and 6, presumed to participate in intramolecular hydrogen bonding, display longer half-lives of 212 and 231 min, respectively, consistent with enhanced protection from solvent exchange.

**References:**

[1] B. A. Johnson, R. A. Blevins, J. Biomol. NMR 1994, 4, 603.

[2] Wüthrich, K. NMR of Proteins and Nucleic acids; Wiley-Interscience: New York, 1986.

[3] D.A. Case, P.A. Kollman et al. (2022), Amber 2022, University of California, San Francisco.

[4] Gaussian 09, Revision D.01, M. J. Frisch, et al., Gaussian, Inc., Wallingford, CT: 2013.

[5] R. Koradi, M. Billeter, K. Wuthrich, J. Mol. Graph. 1996, 14, 51.

# VI. FTIR studies

Middle-infrared experiments (700-4000 cm-1) were recorded in the transmission mode. The measurements were carried out on a Bruker Tensor 27 spectrometer equipped with a deuterated *(L)*-alanine doped triglycene sulphate (DLATGS) pyroelectric detector, a Globar source, and potassium bromide (KBr) beam splitter. The spectral resolution was 4 cm^-1^ and 128 scans were co-added for each spectrum. The compounds were dissolved at 2 or 5 mM in CHCl_3_ and transferred in a liquid cell equipped with CaF_2_ windows (500 μm). The FTIR spectra were not smoothed. Estimations of positions and contributions of discrete subcomponent absorptions have been achieved using curve-fitting approaches with Gaussian functions using OriginPro 2024b (OriginLab Corporation, Northampton, MA, USA). For each compound the experimental spectrum (in black), the deconvolution using a sum of Gaussian functions (NH_2_ in light blue, Free NH in blue, bond NH in light purple, Free CO in purple and bond CO in pink) and the overall fit (in red) are shown.

Figure S15: Amide A and amide I regions of FTIR spectra of oligomers **1a**-**f** at 1 mM in CHCl_3_ at 20°C.

Figure S16: Deconvolution of the ν(NH) and ν(CO) regions of the FTIR spectrum of compound **1a** (2 mM) in CHCl_3_ at 20°C. Amide A (ν(NH)) and amide I (ν(CO)) regions are shown in the left and right panels, respectively.

Table S24: Band positions, band widths and band areas for compound **1a**.

| Absorption bands | Wavenumbers (cm^-1^) | Width (a.u.) | Area (a.u.) |
| --- | --- | --- | --- |
| 1 (NH_2_) | 3517.69 | 21.69 | 0.56 |
| 2 (Free NH) | 3394.69 | 21.50 | 0.49 |
| 3 (Bound NH) | 3330.61 | 122.23 | 1.68 |
| 4 (Free CO) | 1678.57 | 17.69 | 2.20 |
| 5 (Bound CO) | 1657.40 | 26.00 | 2.74 |

Figure S17: Deconvolution of the ν(NH) and ν(CO) regions of the FTIR spectrum of compound **1b** (2 mM) in CHCl_3_ at 20°C. Amide A (ν(NH)) and amide I (ν(CO)) regions are shown in the left and right panels, respectively.

Table S25: Band positions, band widths and band areas for compound **1b**.

| Absorption bands | Wavenumbers (cm^-1^) | Width (a.u.) | Area (a.u.) |
| --- | --- | --- | --- |
| 1 (NH_2_) | 3517.69 | 21.69 | 0.56 |
| 2 (Free NH) | 3392.95 | 26.51 | 0.76 |
| 3 (Bound NH) | 3326.21 | 135.59 | 2.97 |
| 4 (Free CO) | 1677.72 | 19.50 | 2.54 |
| 5 (Bound CO) | 1656.66 | 29.11 | 4.67 |

Figure S18: Deconvolution of the ν(NH) and ν(CO) regions of the FTIR spectrum of compound **1c** (2 mM) in CHCl_3_ at 20°C. Amide A (ν(NH)) and amide I (ν(CO)) regions are shown in the left and right panels, respectively.

Table S26: Band positions, band widths and band areas for compound **1c**.

| Absorption bands | Wavenumbers (cm^-1^) | Width (a.u.) | Area (a.u.) |
| --- | --- | --- | --- |
| 1 (NH_2_) | 3517.88 | 22.70 | 0.63 |
| 2 (Free NH) | 3390.73 | 30.89 | 1.41 |
| 3 (Bound NH) | 3321.82 | 129.06 | 5.43 |
| 4 (Free CO) | 1673.43 | 23.33 | 5.91 |
| 5 (Bound CO) | 1652.67 | 24.47 | 6.21 |

Figure S19: Deconvolution of the ν(NH) and ν(CO) regions of the FTIR spectrum of compound **1d** (2 mM) in CHCl_3_ at 20°C. Amide A (ν(NH)) and amide I (ν(CO)) regions are shown in the left and right panels, respectively.

Table S27: Band positions, band widths and band areas for compound **1d**.

| Absorption bands | Wavenumbers (cm^-1^) | Width (a.u.) | Area (a.u.) |
| --- | --- | --- | --- |
| 1 (NH_2_) | 3517.88 | 20.73 | 0.55 |
| 2 (Free NH) | 3390.10 | 31.62 | 1.81 |
| 3 (Bound NH) | 3321.89 | 124.88 | 6.14 |
| 4 (Free CO) | 1671.78 | 23.54 | 7.42 |
| 5 (Bound CO) | 1651.68 | 24.75 | 7.56 |

Figure S20: Deconvolution of the ν(NH) and ν(CO) regions of the FTIR spectrum of compound **1e** (2 mM) in CHCl_3_ at 20°C. Amide A (ν(NH)) and amide I (ν(CO)) regions are shown in the left and right panels, respectively.

.

Table S28: Band positions, band widths and band areas for compound **1e**.

| Absorption bands | Wavenumbers (cm^-1^) | Width (a.u.) | Area (a.u.) |
| --- | --- | --- | --- |
| 1 (NH_2_) | 3518.50 | 21.06 | 0.54 |
| 2 (Free NH) | 3389.92 | 34.34 | 2.37 |
| 3 (Bound NH) | 3317.33 | 129.51 | 8.25 |
| 4 (Free CO) | 1671.81 | 23.06 | 8.87 |
| 5 (Bound CO) | 1651.65 | 24.02 | 8.95 |

Figure S21: Deconvolution of the ν(NH) and ν(CO) regions of the FTIR spectrum of compound **1f** (2 mM) in CHCl_3_ at 20°C. Amide A (ν(NH)) and amide I (ν(CO)) regions are shown in the left and right panels, respectively.

Table S29: Band positions, band widths and band areas for compound **1f**.

| Absorption bands | Wavenumbers (cm^-1^) | Width (a.u.) | Area (a.u.) |
| --- | --- | --- | --- |
| 1 (NH_2_) | 3518.30 | 22.20 | 0.60 |
| 2 (Free NH) | 3389.22 | 35.32 | 2.97 |
| 3 (Bound NH) | 3315.95 | 132.83 | 10.50 |
| 4 (Free CO) | 1672.00 | 23.98 | 11.05 |
| 5 (Bound CO) | 1651.70 | 25.11 | 11.45 |

Figure S22: Deconvolution of the ν(NH) and ν(CO) regions of the FTIR spectrum of compound **2a** (2 mM) in CHCl_3_ at 20°C. Amide A (ν(NH)) and amide I (ν(CO)) regions are shown in the left and right panels, respectively.

Table S30: Band positions, band widths and band areas for compound **2a**.

| Absorption bands | Wavenumbers (cm^-1^) | Width (a.u.) | Area (a.u.) |
| --- | --- | --- | --- |
| 1 (NH_2_) | 3520.10 | 20.33 | 0.34 |
| 2 (Free NH) | 3402.40 | 19.47 | 0.35 |
| 3 (Bound NH) | 3301.02 | 145.99 | 1.24 |
| 4 (Free CO) | 1680.58 | 14.51 | 0.59 |
| 5 (Bound CO) | 1665.56 | 40.81 | 2.30 |
| 6 | 1619.66 | 15.29 | 0.91 |
| 7 | 1601.17 | 0.36 | 14.25 |

Figure S23: Deconvolution of the ν(NH) and ν(CO) regions of the FTIR spectrum of compound **2b** (2 mM) in CHCl_3_ at 20°C. Amide A (ν(NH)) and amide I (ν(CO)) regions are shown in the left and right panels, respectively.

Table S31: Band positions, band intensities, band widths and band areas for compound **2b**.

| Absorption bands | Wavenumbers (cm^-1^) | Width (a.u.) | Area (a.u.) |
| --- | --- | --- | --- |
| 1 (NH_2_) | 3520.10 | 20.33 | 0.34 |
| 2 (Free NH) | 3401.71 | 23.07 | 0.55 |
| 3 (Bound NH) | 3297.37 | 145.49 | 2.38 |
| 4 (Free CO) | 1680.12 | 16.67 | 0.94 |
| 5 (Bound CO) | 1657.59 | 35.15 | 2.81 |
| 6 | 1620.13 | 1.80 | 15.77 |
| 7 | 1599.45 | 16.23 | 0.50 |

Figure S24: Deconvolution of the ν(NH) and ν(CO) regions of the FTIR spectrum of compound **2c** (2 mM) in CHCl_3_ at 20°C. Amide A (ν(NH)) and amide I (ν(CO)) regions are shown in the left and right panels, respectively.

Table S32: Band positions, band widths and band areas for compound **2c**.

| Absorption bands | Wavenumbers (cm^-1^) | Width (a.u.) | Area (a.u.) |
| --- | --- | --- | --- |
| 1 (NH_2_) | 3520.10 | 20.33 | 0.34 |
| 2 (Free NH) | 3402.49 | 24.66 | 0.78 |
| 3 (Bound NH) | 3297.05 | 147.69 | 4.16 |
| 4 (Free CO) | 1679.52 | 17.06 | 1.09 |
| 5 (Bound CO) | 1658.34 | 34.10 | 6.51 |
| 6 | 1620.20 | 16.65 | 3.64 |
| 7 | 1598.65 | 18.14 | 1.15 |

Figure S25: Deconvolution of the ν(NH) and ν(CO) regions of the FTIR spectrum of compound **2d** (2 mM) in CHCl_3_ at 20°C. Amide A and amide I regions are shown in the left and right panels, respectively.

Table S33: Band positions, band widths and band areas for compound **2d**.

| Absorption bands | Wavenumbers (cm^-1^) | Width (a.u.) | Area (a.u.) |
| --- | --- | --- | --- |
| 1 (NH_2_) | 3520.10 | 20.33 | 0.34 |
| 2 (Free NH) | 3402.52 | 26.67 | 1.02 |
| 3 (Bound NH) | 3296.12 | 149.81 | 5.86 |
| 4 (Free CO) | 1679.11 | 15.54 | 0.89 |
| 5 (Bound CO) | 1658.42 | 34.51 | 9.07 |
| 6 | 1620.23 | 16.78 | 4.97 |
| 7 | 1598.65 | 18.74 | 1.82 |

Figure S26: Deconvolution of the ν(NH) and ν(CO) regions of the FTIR spectrum of compound **2e** (2 mM) in CHCl_3_ at 20°C Amide A (ν(NH)) and amide I (ν(CO)) regions are shown in the left and right panels, respectively.

Table S34: Band positions, band widths and band areas for compound **2e**.

| Absorption bands | Wavenumbers (cm^-1^) | Width (a.u.) | Area (a.u.) |
| --- | --- | --- | --- |
| 1 (NH_2_) | 3520.10 | 20.33 | 0.3413 |
| 2 (Free NH) | 3402.83 | 27.55 | 1.2006 |
| 3 (Bound NH) | 3295.95 | 144.66 | 7.34955 |
| 4 (Free CO) | 1679.23 | 17.12 | 1.16248 |
| 5 (Bound CO) | 1657.85 | 33.44 | 11.09019 |
| 6 | 1620.18 | 16.97 | 6.47443 |
| 7 | 1598.02 | 18.56 | 2.12001 |

Figure S27: Deconvolution of the ν(NH) and ν(CO) regions of the FTIR spectrum of compound **2f** (2 mM) in CHCl_3_ at 20°C. Amide A (ν(NH)) and amide I (ν(CO)) regions are shown in the left and right panels, respectively.

Table S35: ν(NH) and ν(CO) Band positions, band widths and band areas for compound **2f**.

| Absorption bands | Wavenumbers (cm^-1^) | Width (a.u.) | Area (a.u.) |
| --- | --- | --- | --- |
| 1 (NH_2_) | 3520.28 | 19.39 | 0.37 |
| 2 (Free NH) | 3403.38 | 28.11 | 1.33 |
| 3 (Bound NH) | 3294.22 | 147.15 | 7.88 |
| 4 (Free CO) | 1679.96 | 17.16 | 1.15 |
| 5 (Bound CO) | 1658.10 | 34.68 | 13.30 |
| 6 | 1620.04 | 16.90 | 7.09 |
| 7 | 1597.19 | 17.73 | 2.20 |

Table S36: Percentages of free and H-bonded NH and CO of oligomers **1a-f** in chloroform. Comparison with the theoretical percentages calculated from the helix alternating extended and C7 conformations Ext/7-Helix, and the 7-Helix.

| **Compounds** | CO area | Free CO  area | Bound CO  area | Experimental bound CO  (%) | Calculated bound CO in 7-Helix  (%) | Calculated bound CO in Ext/7-Helix  (%) |
| --- | --- | --- | --- | --- | --- | --- |
| **1a** | 4.94 | 2.20 | 2.74 | 56 | 67 | 33 |
| **1b** | 7.21 | 2.54 | 4.67 | 65 | 80 | 40 |
| **1c** | 12.13 | 5.91 | 6.22 | 51 | 89 | 43 |
| **1d** | 14.98 | 7.42 | 7.56 | 50 | 89 | 44 |
| **1e** | 17.81 | 8.87 | 8.94 | 50 | 91 | 45 |
| **1f** | 22.50 | 11.05 | 11.45 | 51 | 92 | 46 |

Table S37: Percentages of free and H-bonded CO areas, of oligomers **2a-f** in chloroform. Comparison with the theoretical percentages calculated from the helix alternating extended and C7 turns (Ext/7-Helix), and the 7-Helix.

| **Compounds** | CO total area | Free CO  area | Bound CO  area | Experimental bound CO  (%) | Calculated bound CO in 7-Helix  (%) | Calculated bound CO in Ext/7-Helix  (%) |
| --- | --- | --- | --- | --- | --- | --- |
| **2a** | 2.90 | 0.60 | 2.30 | *80* | *67* | 33 |
| **2b** | 3.75 | 0.94 | 2.81 | *75* | *80* | 40 |
| **2c** | 7.60 | 1.09 | 6.51 | *86* | *89* | 43 |
| **2d** | 9.96 | 0.89 | 9.07 | *91* | *89* | 44 |
| **2e** | 12.26 | 1.16 | 11.10 | *91* | *91* | 45 |
| **2f** | 14.45 | 1.15 | 13.30 | *92* | *92* | 46 |

# VII. Circular dichroism

Circular dichroism (CD) experiments were carried out using a Jasco J815 spectropolarimeter. The spectra were obtained in CHCl_3_ using a 1 mm path length CD cuvette, at 20°C, over a wavelength range of 190-400 nm. Continuous scanning mode was used, with a response of 1.0 s with 0.1 nm steps and a bandwidth of 1 nm. The signal to noise ratio was improved by acquiring each spectrum over an average of two scans. Baseline was corrected by subtracting the background from the sample spectrum. The samples were typically dissolved at 100-200 µM, depending on oligomer length.

# VIII. 2D NMR spectra of **1a-b**, **1g** and **2a-c**.

COSY NMR spectrum of **1a** in chloroform at 298 K

TOCSY NMR spectrum of **1a** in chloroform at 298 K

ROESY NMR spectrum of **1a** in chloroform at 298 K

^15^N-HSQC NMR spectrum of **1a** in chloroform at 298 K

^13^C-HSQC NMR spectrum of **1a** in chloroform at 298 K

^13^C-HMBC NMR spectrum of **1a** in chloroform at 298 K

COSY NMR spectrum of **1b** in chloroform at 298 K

TOCSY NMR spectrum of **1b** in chloroform at 298 K

ROESY NMR spectrum of **1b** in chloroform at 298 K

^15^N-HSQC NMR spectrum of **1b** in chloroform at 298 K

^13^C-HSQC NMR spectrum of **1b** in chloroform at 298 K

^13^C-HMBC NMR spectrum of **1b** in chloroform at 298 K

COSY NMR spectrum of **1g** in chloroform at 298 K

TOCSY NMR spectrum of **1g** in chloroform at 298 K

ROESY NMR spectrum of **1g** in chloroform at 298 K

^15^N-HSQC NMR spectrum of **1g** in chloroform at 298 K

^13^C-HSQC NMR spectrum of **1g** in chloroform at 298 K

^13^C-HMBC NMR spectrum of **1g** in chloroform at 298 K

COSY NMR spectrum of **2a** in chloroform at 298 K

TOCSY NMR spectrum of **2a** in chloroform at 298 K

ROESY NMR spectrum of **2a** in chloroform at 298 K

^15^N-HSQC NMR spectrum of **2a** in chloroform at 298 K

^13^C-HSQC NMR spectrum of **2a** in chloroform at 298 K

^13^C-HMBC NMR spectrum of **2a** in chloroform at 298 K

COSY NMR spectrum of **2b** in chloroform at 243 K

TOCSY NMR spectrum of **2b** in chloroform at 243 K

ROESY NMR spectrum of **2b** in chloroform at 243 K

^15^N-HSQC NMR spectrum of **2b** in chloroform at 243 K

^13^C-HSQC NMR spectrum of **2b** in chloroform at 243 K

^13^C-HMBC NMR spectrum of **2b** in chloroform at 243 K

COSY NMR spectrum of **2c** in chloroform at 243 K

TOCSY NMR spectrum of **2c** in chloroform at 243 K

ROESY NMR spectrum of **2c** in chloroform at 243 K

^15^N-HSQC NMR spectrum of **2c** in chloroform at 243 K

^13^C-HSQC NMR spectrum of **2c** in chloroform at 243 K

^13^C-HMBC NMR spectrum of **2c** in chloroform at 243 K

COSY NMR spectrum of **2d** in chloroform at 253 K

TOCSY NMR spectrum of **2d** in chloroform at 253 K

ROESY NMR spectrum of **2d** in chloroform at 253 K

^15^N-HSQC NMR spectrum of **2d** in chloroform at 253 K

^13^C-HSQC NMR spectrum of **2d** in chloroform at 253 K

^13^C-HMBC NMR spectrum of **2d** in chloroform at 253 K

# IX. MS-spectra

Compound **1a**:

Compound **1b**:

Compound **1c**:

Compound **1d**:

Compound **1e**:

Compound **1f**:

Compound **1g**:

Compound **2a**:

Compound **2b**:

Compound **2c**:

Compound **2d**:

Compound **2e**:

Compound **2f**:
